# Comparing Learning Outcomes and Student and Instructor Perceptions of a Simultaneous Online versus In-Person Biochemistry Laboratory Course

**DOI:** 10.1021/acs.jchemed.3c00571

**Published:** 2024-02-05

**Authors:** Laura Rowe

**Affiliations:** Department of Chemistry, Eastern Kentucky University, Richmond, Kentucky 40475, United States

**Keywords:** Upper-Division Undergraduate, Biochemistry, Laboratory Instruction, Internet/Web-Based Learning, Problem Solving/Decision Making, Proteins/Peptides

## Abstract

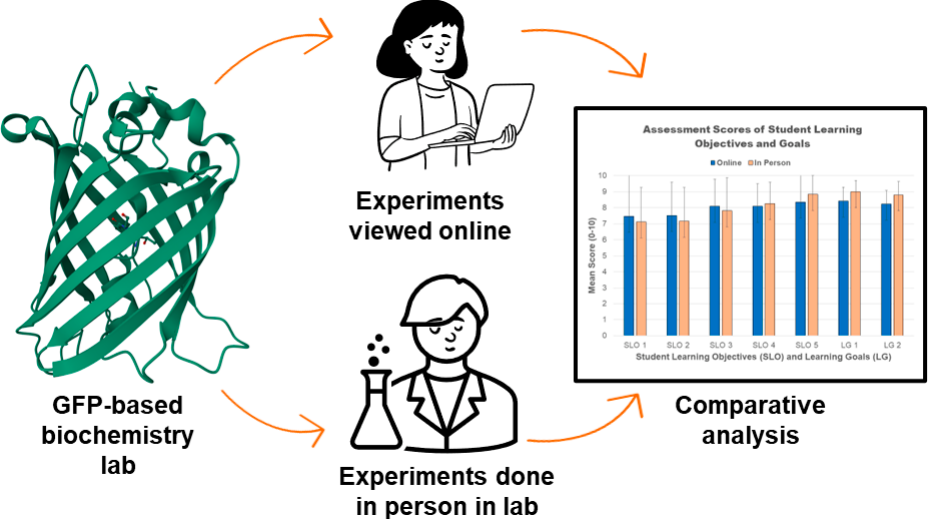

This article compares the learning outcomes and student perceptions of a one semester undergraduate biochemistry laboratory course that was taught using either a fully online or a fully in-person teaching modality. The semester long biochemistry laboratory mimicked the work sequence a researcher would encounter when transforming a plasmid containing a gene for a recombinant protein (superfolder green fluorescent protein, sf-GFP) and then purifying, identifying, and characterizing that protein. The two modalities of the course were completed in the same semester, by the same instructor, in which students self-selected into which modality they preferred at the beginning of the semester. Students in the in-person section reported enjoying the laboratory course more than the online cohort of students and found it to be less time-consuming. Additionally, a survey of biochemistry laboratory instructors from across the United States, who had experience teaching both online and in-person biochemistry laboratories, indicated that the majority of instructors that responded to the survey preferred the in-person modality: believing them to be more effective and engaging for the students, more enjoyable, and less time-consuming for the instructor. Statistical analysis of formative and summative assessments indicated no significant difference in non-hands-on student learning objective and learning goal scores between the two groups, but the small number of students and instructors in this study limits the generalizability of these results.

## Introduction

Teaching various chemistry courses using an online modality has been a common practice for many years. However, prior to the COVID-19 pandemic, it was much less common to teach chemistry laboratories through a purely online modality, other than a few nonmajors and introductory chemistry courses.^1^ One of the primary purposes of chemistry laboratories is to teach students how to use laboratory equipment and garner hands-on experience, leading many chemistry educators to believe that chemistry laboratories, especially upper-division laboratories (such as biochemistry), should remain in-person.^2−4^ However, COVID-19 guidelines in different nations and educational institutions forced a large majority of biochemistry laboratories to become online-only, or a hybrid of online and in-person, for multiple years.^5^

This article evaluates the different learning outcomes of 2 cohorts of students simultaneously taking an upper-division biochemistry laboratory course and describes the format of the superfolder green fluorescent protein (sf-GFP)-based, one semester biochemistry laboratory that we developed just prior to the pandemic that was utilized during this study. The comparison of the results from the two groups is significant and unique as compared to other published online versus in-person laboratory studies because only one variable was altered during the course delivery: students performed the experimental tasks in-person or watched another student complete the same experimental tasks in an online video. All other significant variables were the same for online versus in-person laboratory cohorts; all students took the course the same semester (Fall 2020), with the same laboratory and lecture instructor, and both cohorts received the same instruction in the lab and lecture (all students were concomitantly taking the same biochemistry lecture course). Additionally, student and instructor perception surveys were collected and assessed in order to determine whether students and instructors preferred one modality over the other or perceived different outcomes between the two different types of laboratory instruction.

## Background

### Laboratory Purpose and Models

The primary purpose of laboratories in chemistry and biochemistry education has long been debated by faculty and chemistry educators, although most can agree that psychomotor (hands-on “doing”) and cognitive (thinking) domains of learning are of primary importance in the laboratory experience.^2,6−9^ Many educators believe that the hands-on practice with equipment, reagents, and methods that students acquire in the lab is essential to understanding that chemistry is something you *do*, and not just something you think about, and is also important experience for future job-seekers.^2^ Likewise, many students also value this role of laboratory in their education, finding the psychomotor, hands-on experience with methods and instruments they may use in their future jobs the greatest asset of laboratory modules.^8^ From this psychomotor-valuing perspective, online only laboratories clearly have a disadvantage as compared to in-person laboratories. However, the cognitive domains of the laboratory experience can be addressed in either an online or an in-person format.

For in-person chemistry and biochemistry laboratories, there are two general laboratory design strategies: (1) “Cookbook” laboratories, and (2) inquiry-based or authentic research-based laboratories.^3,9^ Cookbook laboratories are stand-alone laboratories that give explicit procedural details for a single experiment, which is usually completed in 1 or 2 lab sessions and has the student repeat a well-known experimental procedure that often highlights the validity of a theory discussed in lectures. These laboratories offer the advantages of independent modularity, in that subsequent experiments do not depend on the results of previous experiments. Additionally, the results are comparatively reproducible and there are many commercially available lab manuals and assessments that streamline implementation and assessment among multisection laboratories with multiple instructors. Cookbook laboratories can have the disadvantages of promoting rote learning over meaningful learning and not exposing students to the open-ended nature of scientific research.^3,9,10^

Inquiry-based laboratories and authentic research-based laboratories focus more on an open-ended learning experience, in which there are multiple ways to solve the problem or answer the question. Inquiry-based laboratories pose problems, or ask questions (which often have multiple solutions already known), and guide students to find their own solutions via experimental methods, whereas authentic research-based laboratories require students to work on testing a hypothesis or trying to solve a problem that does not already have a solution or answer.^9−11^ These types of laboratories are often preferred by chemistry education experts due to their ability to promote meaningful learning, help students identify as scientists, and practice science the way it is usually practiced outside of the classroom.^9,10^ However, this format of lab is not always (or even usually) adopted at all colleges or universities due to the disadvantages, which I believe include an increased “messiness” and the difficulty in scaling them up for lab sections with large numbers of students that are led by novice teaching assistants. Additionally, the open-ended nature of many of the questions can make unbiased assessment challenging and time-consuming. Some students also dislike the open-ended nature of these lab formats because the procedures and assessments can lack clarity and certainty and require a degree of autonomy they may not be accustomed to.^9^

The laboratory we developed and used in this study combined some aspects of an authentic course-based undergraduate research experience (CURE) with some explicit procedural steps and well-established procedures for a particular protein purification to develop a biochemistry laboratory with a highly structured CURE format. In many biochemistry research laboratories, researchers must express a desired protein in a recombinant organism (bacteria, yeast, or mammalian cells), and to do so, they transform a plasmid containing the gene of their desired recombinant protein into their cell line, express the protein with antibiotics and inducers, purify the protein, and then confirm the identity of and characterize their protein of interest. This is an essential step in most protein-based biochemistry *in vitro* research since the purified protein must be obtained before other subsequent experiments can be completed. Additionally, scientific training in graduate school and beyond often combines formal written procedures found in protocols and papers with a great deal of less formalized training. This more informal training is often in the form of a senior student or mentor verbally explaining and demonstrating procedures and instrument use and relying on the learner or trainer to take their own thorough notes so they can later replicate the procedure/method independently. Therefore, in our hybrid laboratory, students first determined if an *Escherichia coli* (*E. coli*) cell line contained the correct plasmid and then expressed, purified, and characterized that recombinant protein (superfolder-green fluorescent protein, sf-GFP) using a combination of formal written protocols and informal verbal descriptions and demonstrations.

## Laboratory Course Design

**Figure 1:**
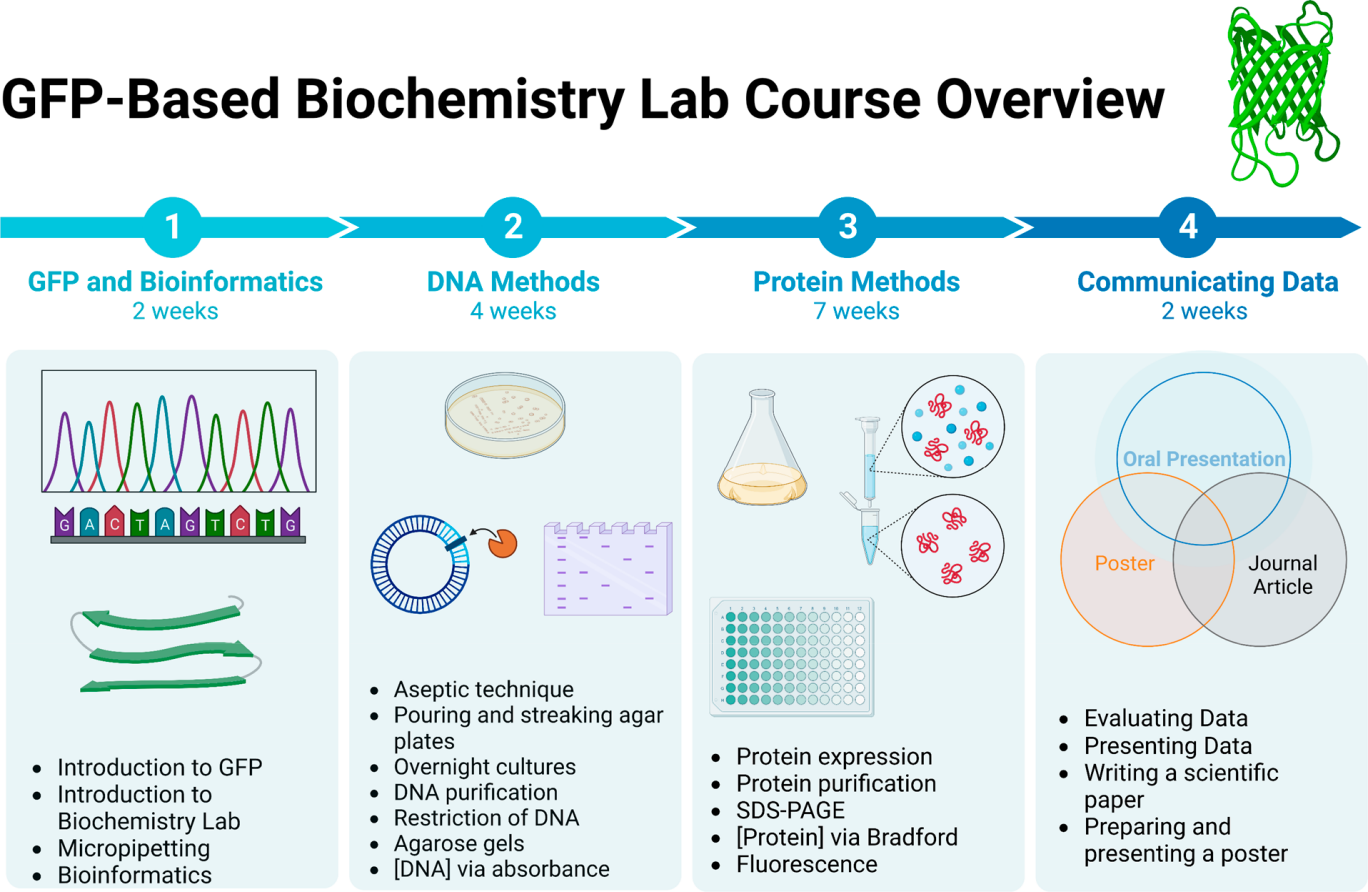
The sf-GFP-based laboratory was sequential in that students in both laboratory sections completed introductory readings, safety information, micropipette training, and a GFP-based bioinformatics laboratory the first 2 weeks of lab. (1) This was followed by 4 weeks of various DNA methods in which the sf-GFP plasmid was purified and identity was confirmed with restriction mapping. (2) Students then expressed, purified, and characterized sf-GFP using protein biochemistry methods (3) and communicated the results of their semester-long lab by designing and orally presenting a poster and writing a JACS-style manuscript (4). Figure created with Biorender.com.

We used a well-characterized and frequently used protein, the superfolder green fluorescent protein (sf-GFP), expressed recombinantly in *E. coli* for this laboratory (see Figure 1).^12^ GFP is used ubiquitously in life science research and has been previously used in undergraduate biochemistry laboratory courses, and the superfolder variant has the advantage of folding into a fluorescently active conformation over a wide-range of chemical and physical conditions, thus making it harder for the students to “kill” (aka denature) during laboratory procedures.^12−16^

The workflow during the semester is summarized in Figure 1. Students completed introductory readings and a bioinformatics laboratory the first 2 weeks of lab. The first formative assessment covering bioinformatics was excluded from assessment since both sections completed it independently outside of laboratory time.^17^ Students then were trained (in-person) or observed a video (online) to use micropipettes, toured the biochemistry lab equipment, learned safety rules, and reviewed important equations. This was followed by 4 weeks of DNA techniques which included aseptic technique using a laminar flow hood and an autoclave, preparing, pouring, and streaking agar plates with *E. coli* cells, and picking colonies to grow in an overnight culture (Lab Report 1). Next, students grew overnight cultures of their cells transformed with a sf-GFP gene containing plasmid, used a commercially available kit to purify the plasmid DNA, quantified plasmid DNA concentration using a Nanodrop instrument and absorbance at 260 nm, linearized their purified plasmid DNA with appropriate restriction enzymes, and assessed fragment size with agarose gel electrophoresis methods (Lab Report 2).^18^

Students next expressed and collected sf-GFP by inoculating overnight cultures in appropriate media, monitoring cell growth with absorbance at 600 nm, and centrifuging the cells. The cell pellet then underwent multiple freeze–thaw cycles to break open cells, and a gravity IMAC affinity column was used to purify the sf-GFP that contained a cleavable 6xHis-tag.^19^ Protein purity was confirmed with SDS-PAGE, while protein concentration was determined with both absorbance and the Bradford assay.^20^ Lastly, the fluorescence emission was collected and analyzed from the purified sf-GFP samples (Lab Report 3). In the final 2 weeks of the lab, the focus shifted on presenting and communicating the result of the students’ semester-long project via visual, written, and oral communication methods. Each student group prepared an ACS-style poster of their lab results and presented it to the class (in-person or online), and each individual student summarized the entire semester’s laboratory in the style of a JACS research manuscript as their final lab report, which served as their summative assessment.

## Student Composition and Institutional Setting

The institution setting (Valparaiso University) was a small (2700 students), private regional university primarily serving undergraduate students. Students taking this laboratory course were third or fourth year undergraduate students who were concomitantly taking the introductory biochemistry lecture course. Students varied in their majors, with a majority being chemistry or biology majors and many having a premed emphasis, and there was a fairly even distribution of males and females. Prerequisites for the course were passing grades in two semesters of general chemistry lecture and lab and two semesters of organic chemistry with lab. A completed semester of analytical chemistry and a semester of physics was suggested but not required to take the course. A total of 24 students participated in both the lecture and lab, with all students attending the same lecture section and student’s self-selecting into either the in-person laboratory section (7 students) or the online-only laboratory section (17 students). One student started out in the in-person section but switched to the online section in the middle of the semester, and their assessments were dropped from subsequent analysis. Moreover, all but two students attended the lecture sections in-person (22 out of 24 students). The two students who did not attend the lectures in-person due to COVID-19 concerns did so in an online capacity by watching the lecture synchronously via Zoom, and both of these students were in the online laboratory section as well.

Students self-selected into either the in-person or online laboratory module due to class scheduling issues or concerns about COVID-19 infection. Both modality offerings were required due to COVID-19 concerns, and the institutions desire to offer as many in-person courses as possible during the pandemic. Since both modalities were required because of pandemic issues, students were able to self-select their modality, and assessments were identical in both modalities; the assessments and variation in course design (in-person versus online) were within the normal requirements of the course, such that the research qualified for IRB exemption under Category 1, in that the research was conducted in established or commonly accepted educational settings that specifically involve normal educational practices that are not likely to adversely impact students’ opportunity to learn required educational content or the assessment of educators who provide instruction. Moreover, all surveys were voluntary and anonymous, and students were informed that the results between the two different modalities were analyzed in a research study prior to survey completion. The instructor survey also qualified for IRB exemption under Category 2, in that it was research that included interactions involving only a survey procedure in which the identity of the human subjects (survey respondents) could not be readily ascertained.

## Results and Discussion

### Biochemistry and Online Laboratories Background

Before the COVID-19 pandemic, there were many opportunities for students to take online chemistry and biochemistry lecture courses but fewer opportunities to take purely online chemistry laboratory courses. Some online programs offered intensive 1–4 week summer lab experiences for upper division chemistry courses for students completing all of their lectures online instead of making their laboratory courses online. The rationale for not offering upper-division laboratory courses purely online was due to the acceptance in the chemistry education community that hands-on laboratory experience is essential for students to gain real-world experience handling scientific equipment and reagents, that the laboratory educational experience is an implicit part of a chemist and biochemist’s identity, and that the laboratory is the place where students learn how to do chemistry (and biochemistry).^2^ Highlighting the importance of the laboratory experience, The American Chemical Society Committee on Professional Training requires bachelor degree chemists to complete 400 h of laboratory experience prior to obtaining ACS certification, while the Royal Society of Chemistry requires 300 h of laboratory work for a bachelor’s degree in chemistry.

However, both of these accrediting bodies and those strongly against purely online chemistry laboratories had to pivot and accept online laboratory modalities during the COVID-19 pandemic. Several recent papers have described online biochemistry laboratory courses that were developed and deployed during COVID-19 shut downs, including virtual biochemistry laboratories, “choose your own adventure” online biochemistry laboratories, project-based biochemistry laboratories that could be completed outside of a traditional lab, and pivoting in-person biochemistry laboratories to online via the use of synchronous and asynchronous video modalities.^21−32^ However, to the best of my knowledge, there have been no studies published comparing student learning outcomes in which the exact same biochemistry laboratory course was simultaneously completed by 2 self-selected cohorts of students, one cohort entirely online and the other entirely in-person, in which all students were taught by the same instructor for the laboratory course.

### Rationale for Laboratory Design

**Figure 2:**
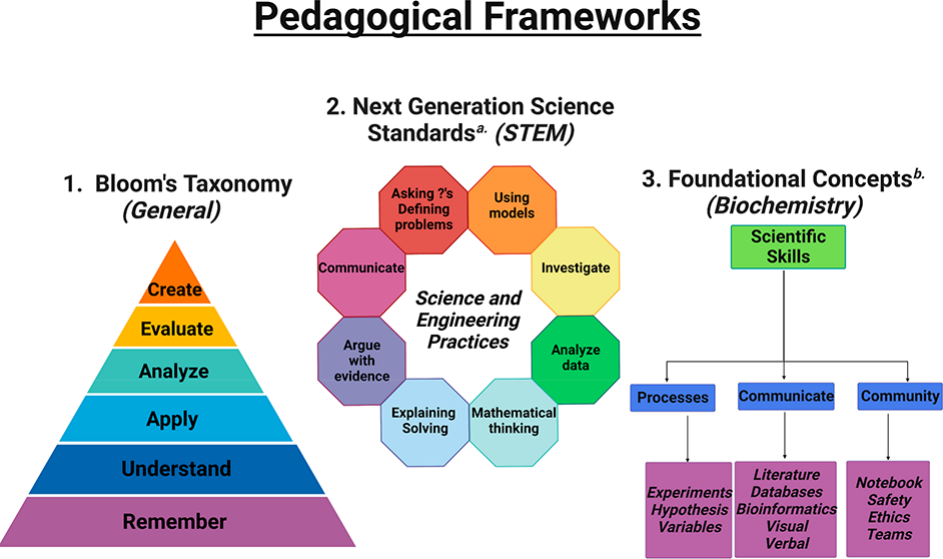
Three pedagogical frameworks used in designing the laboratory and the student learning objectives and learning goals assessed in this study. From general (Bloom’s) to science-specific (NGSS)^a^ to biochemistry-specific (Foundational Concepts in Biochemistry),^b^ these three frameworks provided a logical scaffold for designing and assessing this laboratory. Figure created with Biorender.com.

There are far fewer commercially available laboratory manuals for biochemistry laboratories, as compared to the relative wealth of general and organic lab manuals, and the ones available are often not amenable to a once per week, 3 h laboratory session with up to 24 students at a time, as was our biochemistry laboratory format. For this reason, we decided to design our own biochemistry laboratory that would mimic a realistic series of experimental steps that many undergraduate and graduate biochemistry students must complete during their research projects, in a way that is also similar to authentic research experience, while also addressing many of the higher Bloom’s learning outcomes, Next Generation Science Standards developed by the National Research Council, and the Foundational Concepts in Biochemistry developed by the American Society of Biochemistry and Molecular Biology (Figure 2).^33−36^ A generic workflow in an undergraduate research experience often follows this schedule:

- Step 1. Read literature about research project
- Step 2. Be trained by senior lab students in methods and instruments
- Step 3. Perform experiments and analyze and interpret data
- Step 4. Present results in group meetings and conferences
- Step 5. Write up results for manuscript submission

This workflow is ideally in agreement with many of the pedagogical frameworks detailed in Figure 2, highlighting the high value of research experiences. For example, this workflow inherently encompasses all 4 of the top tiers of Bloom’s taxonomy (apply, analyze, evaluate, create), 6 of the 8 Next Generation Science Standards (asking questions and defining problems, investigating, analyzing data, explaining/solving, arguing with evidence, and communicating), and all three subsets of the scientific skills which are a foundational concept in biochemistry (processes, communicate, and community).^34−36^

### Assessing Student Learning Objectives and Learning Goals

Four formative assessments were given (see Laboratory Course Design), requiring students to analyze and interpret data from multiple weeks of experiments as well as research the rationale of many of the experimental steps they had taken. The first formative assessment, Lab Report 1, which was a computer-based bioinformatics laboratory, was not analyzed in this study because all students completed it at home on their computer without any laboratory experimental procedures or additional in-person instruction, obviating a comparison of in-person versus online student performance. The remaining 3 formative assessments and the summative assessments were also completed outside of laboratory time, but the in-person cohort completed all of the experiments described and analyzed in the assessment in-person, while the online cohort watched videos of a teaching assistant completing the experimental procedures. The online laboratory was asynchronous, but the due dates for assessments for both cohorts were the same. Additionally, although the in-person cohort worked with a lab partner during experimental procedures, sharing collected data, all students in both the in-person and online sections were expected to complete all assessments individually.

The 3 formative assessments and the final lab report (summative assessment) were analyzed for student’s success in meeting the student learning objectives (SLOs) and learning goals (LGs) listed in Table 1. All assessments were analyzed using a 10-point rubric (Supporting Information: Assessment Scoring Rubrics), ranking their responses from little to no indication of meeting learning objective/goal (1) to excellent indication of meeting learning objective/goal (10). Five different student learning objectives (SLO 1–5) were identified for the laboratory course, and these were assessed using specific questions in different lab reports (Supporting Information: Lab Report Sheets; Supporting Information: Assessment Scoring Rubrics). As student learning objectives, and not learning goals, these were tasks that students should have been able to complete and could be assessed in a relatively nonbiased manner. For example, for SLO 1, summarizing procedural steps and stating the purpose of each step, the number of correct procedural steps and purpose requested in specific lab report questions were just counted and then assigned the appropriate rubric score according to the total number (Supporting Information: Assessment Scoring Rubrics).^37^ The two learning goals were assessed with the final lab report and were more generalized goals, rather than specific objectives, which can be reflected in the different rubric scoring categories (Supporting Information: Assessment Scoring Rubrics).^38^

**Table 1:** Student Learning Objectives and Learning Goals

SLO/LG#^a^	SLO/LG	Assessment(s)^b^	Bloom’s^c^	NGSS^c^	Foundational Concepts^c^
SLO 1	Summarize procedural steps and state purpose of each step.	LR1, LR3, FR	Remember, Understand	Explaining	Processes: Experiments
SLO 2	Understand purpose of procedural steps.	LR1, LR2, FR	Understand	Explaining	Processes: Experiments
SLO 3	Use resources provided and available online to gather, understand, and explain/state important information about laboratory procedures.	LR1, LR2, LR3	Understand, Apply	Explaining, Investigate, Communicate	Communicate: Literature, Databases
SLO 4	Analyze experimental results and determine “success” of procedures.	LR2, LR3, FR	Analyze, Evaluate	Explaining, Analyze data, Mathematical thinking	Processes: Experiments, Hypothesis, Variables
SLO 5	Be able to read, interpret, and cite literature relevant to the semester laboratory project.	FR	Understand, Apply	Investigate, Argue with evidence, Communicate	Communicate: Literature, Databases
LG 1	Understand the entire semester’s project as a whole research project, specifically how each week’s experiments and results/data were necessary and informed the subsequent steps of the research project.	FR	Remember, Understand, Analyze, Evaluate	Explaining, Investigate, Analyze data, Mathematical thinking, Argue with evidence, Communicate	Processes: Experiments, Hypothesis, Variables; Communicate: Literature, Databases, Bioinformatics, Visual, Verbal
LG 2	Successfully present and interpret the experimental results of the semester in a scientific journal format.	FR	Remember, Understand, Analyze, Evaluate, Create	Explaining, Investigate, Analyze data, Mathematical thinking, Argue with evidence, Communicate	Processes: Experiments, Hypothesis, Variables; Communicate: Literature, Databases, Bioinformatics, Visual, Verbal

a The five assessed student learning objectives (SLO) and two learning goals (LG) for the semester are listed.

b Assessments provided data for those specific SLOs and LGs (LR stands for Lab Report Sheet, and FR stands for Final Lab Report).

c The Bloom’s, NGSS, and Biochemistry Foundational Concepts that specific SLOs and LGs targeted are also summarized.

The SLOs and LGs were designed to target different pedagogical aspects of the three frameworks summarized in Figure 2 and Table 1. Since one cohort of students would be completely online, no “hands-on” skills were identified as a SLO of a LG. Although this hands-on training is undoubtedly one of the most important aspects of a biochemistry laboratory course, the online cohort had no opportunity to practice hands-on activities, nor was the instructor able to assess any hands-on objectives. As summarized in Table 1, the SLOs and LGs targeted all of Bloom’s pedagogical hierarchical levels, all but one (using models) of the NGSS science and engineering practices, and two of the scientific skills (processes and communicate) identified as essential in Foundational Concepts in Biochemistry by the American Society of Biochemistry and Molecular Biology.^34−36^ Future improvements in these assessments could be made by incorporating a 3-dimensional learning framework that consolidates much of the literature as to how students learn science, with the three dimensions including scientific and engineering practices, cross-cutting concepts, and disciplinary core ideas.^39^ Although these assessments focus effectively on scientific and engineering practices, a more thorough incorporation of cross-cutting concepts and scaffolded disciplinary core ideas would be beneficial to student learning.

**Figure 3:**
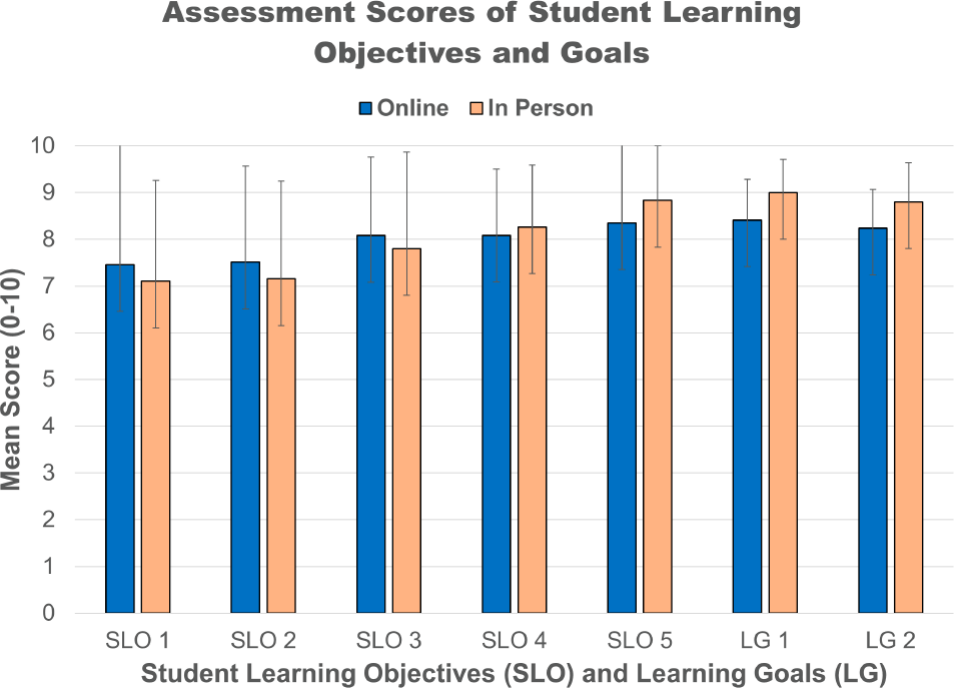
10-point rubric-based scores for each SLO and LG assessed in the 3 formative and 1 summative assessments were combined separately for the students taking the online modality (blue) and the in-person modality (orange), and mean and standard deviations of SLOs and LGs were determined.

The rubric-based scores of each SLO and LG for each student in the online (17 students) and in-person (6 students) sections were then analyzed separately for mean score and standard deviation of each SLO and LG. Figure 3 shows the results, with slight differences in the mean score between the online and in-person cohorts (error bars representing ±1 stdev). From this comparison, in-person students scored higher on SLO 5, LG 1, and LG 2, but online students scored higher on SLO 1, SLO 2, and SLO 3.

Although there are small differences in the mean scores between the online and in-person cohorts, the ±1 stdev error bars have extensive overlap. The two data sets were therefore analyzed to determine if there were any statistical outliers using a Grubb’s test (90%); no data points could be excluded from the online data set, but three data points were excluded from the in-person data set (1 data point in SLO 1 and 1 data point in both LG 1 and LG 2). Following the Grubb’s test and the exclusion of those outliers from further statistical analysis, an F-test (95%) was used to determine if there was statistically any difference between the standard deviations of the two data sets. There was no significant difference in the standard deviations of the two data sets, with the exception of SLO 5, in which *F*_calc_ was greater than *F*_table_. Lastly, a 2 sample *t* test was performed on the data sets, using equal variances for all comparisons (SLO 1, SLO 2, SLO 3, SLO 4, LG 1, LG 2) except SLO 5, which used unequal variances. Results of the *t* test indicated that there was no statistical difference between the means of any of the SLOs or LGs of the online versus the in-person student cohort, with all *p*-values being much greater than 5% in all cases (Supporting Information: Statistical Analysis) However, the small number of students (6) in the in-person cohort and the fact that not all data sets exhibited a normal distribution upon histogram inspection (Supporting Information: Statistical Analysis and Histograms) likely limit the strength of this statistical analysis. Nonetheless, analysis of SLO and LG assessments did not indicate that one laboratory modality resulted in greater achievement in meeting the stated student learning objectives or learning goals, suggesting that for many cognitive, but not hands-on, aspects, an online modality for biochemistry laboratory is equivalent to an in-person laboratory in terms of learning gains.

Although the statistical analysis did not show a significant difference in meeting stated SLOs and LGs in the online versus in-person biochemistry laboratory modalities, the student and instructor perception surveys did show a significant difference. Both students and instructors indicated greater satisfaction with the in-person laboratories and believed that the in-person mode of learning was better for learning and engagement.

## Student Perceptions of Online versus In-Person Modalities

**Figure 4:**
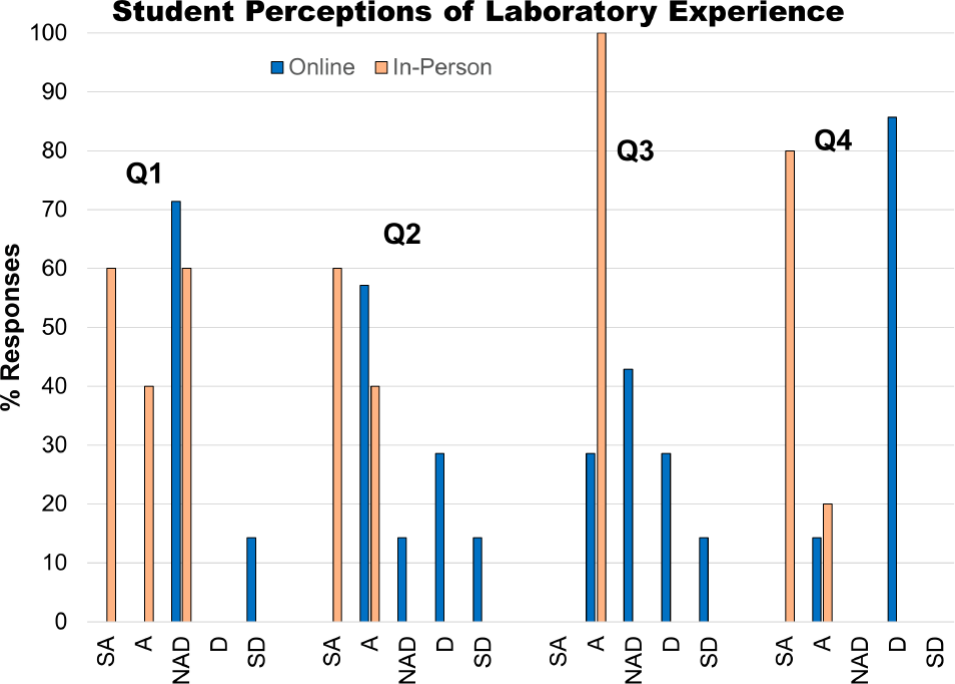
Student perceptions of their laboratory experience via responses to a 5-point Likert scale: SA = strongly agree, A = agree, NAD = neither agree nor disagree, D = disagree, and SD = strongly disagree. Q1 = I understood what was going on in lab. Q2 = I felt engaged completing laboratory work. Q3 = Completing the lab section was useful. Q4 = If I had to choose again, I would choose the same section.

At the end of the course, students completed an anonymous survey concerning their perceptions and experience of the online or in-person biochemistry laboratory sections (Supporting Information: Student Survey; Figure 4). A variety of questions were asked, including ten questions that asked them to reflect on the laboratory experience in general and 13 questions that asked them to assess whether or not the lab helped them meet potential goals/objectives of taking the course. A 5-point Likert scale (strongly agree, agree, neither agree nor disagree, disagree, strongly disagree) was used for both of these question sets. The results of the most relevant general reflection questions are summarized in Figure 4, indicating that a larger percentage of students in the in-person section thought they “knew what was going on in lab”, felt engaged completing lab work, and believed the course was useful. All of the in-person students surveyed indicated that, if they had to choose again, they would choose the in-person lab section over the online section, whereas only 15% of online students would choose the online modality again (Figure 4).

Results asking if the lab was useful in meeting potential learning goals indicated that the online group believed the lab successfully addressed different learning goals than the in-person group. For the online group of students, the top 5 learning goals that the majority of the students agreed that the course successfully met were: (1) To develop my scientific writing skills, (2) To make connections between lab and the real world, (3) To understand how a chemistry research lab works, (4) To connect concepts learned in lectures with laboratories, and (5) To learn how to design and carry out experiments. The in-person cohort identified these as the top 5 learning goals successfully addressed in the lab: (1) To prepare for the career I want to pursue, (2) To learn lab techniques, (3) To prepare for future science courses, (4) To carry out experiments safely, and (5) To apply lab techniques (Supporting Information: Student Survey Results). Interestingly, there was no overlap in the top 5 between the two different cohorts, with online students emphasizing more conceptual skills (writing, making connections) while in-person students identifying more hands-on and career-beneficial goals, such as preparing for their future career and applying lab techniques.

To summarize, a significantly higher percentage of students in the in-person section felt that they understood what was going on in lab, felt engaged during lab work, thought the lab was useful, and would choose to take that modality again than students in the online section (Figure 4). Additionally, both cohorts found their lab experience useful in meeting multiple learning goals, but which goals the lab successfully addressed varied on modality, with in-person students emphasizing career-readiness goals more than online students.

## Instructor Perceptions of Online versus In-Person Modalities

Following the completion of this laboratory, in Fall of 2021 and Spring of 2022, an online survey assessing the perceptions instructors have toward online versus in-person biochemistry laboratories was created using a 5-point Likert scale. The survey consisted of 20 questions, with a balance of positively and negatively biased questions used in order to reduce bias (Table 2, Supporting Information: Instructor Survey and Results). For example, in Question #3 (Table 2), respondents were asked to rank their agreement with the statement “The online only biochemistry laboratory was equivalent to an in-person biochemistry laboratory in terms of student learning” (positive bias toward online modality) and then later were asked to rate their level of agreement to the converse statement “The online only biochemistry laboratory was NOT as effective as an in-person biochemistry laboratory in terms of student learning” (negative bias toward online modality).

**Table 2:** Select Instructor Survey Questions

Question #	Positive Bias Version	Negative Bias Version
Q1	I enjoyed teaching an online only biochemistry laboratory as much as an in-person biochemistry laboratory.	I prefer teaching in-person biochemistry laboratories over online only biochemistry laboratories.
Q2	The online only biochemistry laboratory required LESS time and effort from me, the instructor, than an in-person biochemistry laboratory.	The online only biochemistry laboratory required MORE time and effort from me, the instructor, than an in-person biochemistry laboratory.
Q3	The online only biochemistry laboratory was equivalent to an in-person biochemistry laboratory in terms of student learning.	The online only biochemistry laboratory was NOT as effective as an in-person biochemistry laboratory in terms of student learning.
Q4	The online only biochemistry laboratory was equivalent or as good as an in-person biochemistry laboratory in terms of student engagement and interest.	The online only biochemistry laboratory was NOT equivalent or as good as an in-person biochemistry laboratory in terms of student engagement and interest.

SurveyMonkey was used for the survey construction and distribution, and respondents were incentivized to respond with entry into a drawing (first 50 respondents) to receive a $100 Amazon gift card. One hundred and ten biochemistry instructors were contacted, and they were identified using the primary contact from the ASBMB institution membership and from searching individual college and university Web sites to identify professors and instructors that taught biochemistry laboratories. All instructors were contacted once via a personalized email, and if no response to the email was received and the survey was not completed by them, a follow up email was sent three months later. Of 125 individuals contacted, 19 completed the survey, giving a response rate of 15%.

The distribution of respondents by institution type is shown in Table 3. None of the instructors surveyed had ever taught an online biochemistry laboratory course before the COVID-19 pandemic.

**Table 3:** Percentage of Instructor Survey Respondents by Institution Type

Institution Type^a^	Percentage of Respondents
Large, public	21
Large, private	5
Medium, public	16
Medium, private	16
Small, public	0
Small, private	42
Community	0

a Large public and private institutions are colleges and universities with >15,000 students; medium public and private institutions are colleges and universities with between 5,000 and 15,000 students; small public and private institutions are colleges and universities with <5,000 students.

Due to pandemic restrictions, however, all surveyed instructors had taught at least one online biochemistry laboratory course, and 58% indicated that their online biochemistry laboratory was fully online, whereas 42% taught a hybrid course in which a mixture of online laboratories and in-person laboratories occurred during the course (Supporting Information: Instructor Survey and Results). Forty three percent of respondents used livestream, 29% used prerecorded videos, and 29% used fully digital or lab simulation laboratories. The instructor surveyed was the primary instructor for the laboratory course 89% of the time, whereas the primary instructor was either a teaching assistant or a combination of a teaching assistant and the surveyed instructor 11% of the time (Supporting Information: Instructor Survey and Results).

In terms of institutional and publicly available support, 37% of surveyed instructors agreed with the statement “My institution provided enough support for the change to an online biochemistry laboratory”, while 37% disagreed or strongly disagreed with that statement and 26% neither agreed nor disagreed. Eighty three percent of respondents had to livestream or prerecord their own material for their online biochemistry course because there was not sufficient free, or commercially available, material, with 68% of respondents disagreeing (and 32% neither agreeing nor disagreeing) with the statement “The pre-made digital biochemistry laboratories that were commercially, or freely, available were sufficient for me to effectively design an online biochemistry laboratory.” (Supporting Information: Instructor Survey and Results).

**Figure 5:**
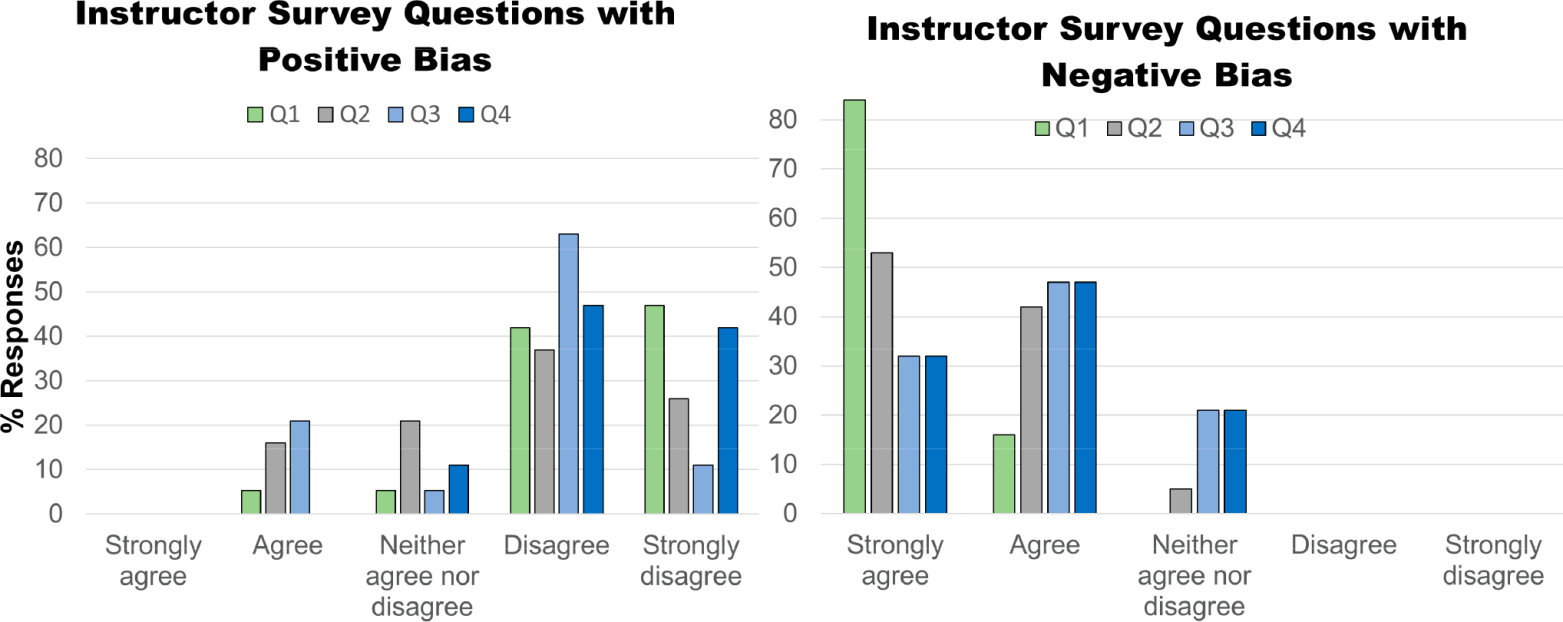
Instructor survey responses to four statements concerning online versus in-person biochemistry laboratories. Questions were asked with both a positive (toward online) and a negative (against online) bias, for a total of 8 questions, shown in Table 2.

The survey also indicated that 100% of instructors surveyed preferred teaching an in-person modality over an online modality for biochemistry laboratories, with 74% of instructors indicating that the online modality also required more time and effort on their part than the in-person modality (Supporting Information: Instructor Survey and Results and Figure 5, Table 2). Ninety five percent of respondents found the in-person laboratory modality to be better for student engagement and comprehension, while 79% did not think an online laboratory format was as effective for student learning as in-person and 89% of instructors would not choose to teach online biochemistry laboratories again (Supporting Information: Instructor Survey and Results and Figure 5, Table 2). However, one useful aspect of the forced online pivot is that a significant percentage of instructors did find some individual online biochemistry modules useful, with 68% stating that they will keep some online biochemistry modules in future in-person biochemistry laboratory courses (Supporting Information: Instructor Survey and Results).

The overwhelming preference of instructors for in-person biochemistry laboratories over an online modality indicated by the survey results may not be generalizable to a more representative sample of instructors for several reasons. First, only 19 of the 125 instructors contacted responded to the survey, and all instructors were based in the U.S. This small number is not large enough to assume population generalizability. Additionally, it may have been difficult for instructors to separate their opinion about online laboratory modalities, which were often forced upon instructors due to local and institutional COVID-19 policies with little to no preparation time or training, from overall negative feelings about living and working through the pandemic.

## Limitations

Although we believe these results are interesting, the small sample size of students in the in-person laboratory modality and instructor survey limits the generalizability of the results. In terms of the two student cohorts, only 7 students self-selected into the in-person laboratory modality, and one had to be excluded from analysis due to them changing to the online format midsemester. A sample size of only six data points is almost certainly not representative of a population, and this small number limits the statistical power of the *t* test comparing the differences in the mean student learning objectives and goal scores between the two populations. The null hypothesis in the *t* test for this study was that there is no statistical difference between the means of the scores of the in-person versus online laboratory students. The results of the *t* test accepted this null hypothesis, with *p*-values well below 5% for all analyzed student learning objectives and learning goals. However, such a small sample size increases the possibility of a Type II error, failing to reject a null hypothesis when we should have. Additionally, since students self-selected into the different modalities, the two populations were not necessarily matched in terms of mastery of previous knowledge and overall academic achievement, although all students were required to have earned a C or better in several prerequisite courses. Likewise, the small number of instructors completing the survey and the newly designed questions in the questionnaire that had not been externally validated limit the generalizability of the results.

## Conclusions

A semester long biochemistry laboratory course based on the expression, purification, and analysis of the recombinant protein sf-GFP was developed to more realistically represent the science as practiced. The laboratory was taught both completely online and completely in-person to two different cohorts of students during the same semester. A comparison of student’s ability to meet seven different student learning objectives/learning goals that were cognitive-based, and not hands-on, objectives did not indicate any significant difference in the learning outcome of the online versus the in-person students. Student and instructor perception surveys both strongly indicated a preference for in-person biochemistry laboratories due to a perception of better learning outcomes and increased student and instructor engagement and enjoyment. However, the small sample size in both the instructor survey and the in-person laboratory cohort limits the generalizability of these results.

## Supporting Information Available

Lab Report Sheets (PDF; DOCX)
Assessment Scoring Rubrics (PDF; DOCX)
Histograms (PDF; DOCX)
Statistical Analysis (XLSX)
Student Survey (PDF; DOCX)
Student Survey Results (XLSX)
Instructor Survey and Results (PDF)
