# Supplementary material for: Comparing Learning Outcomes and Student and Instructor Perceptions of a Simultaneous Online versus In-Person Biochemistry Laboratory Course
Source: J Chem Educ. 2024 Feb 5;101(3):882–91. doi: 10.1021/acs.jchemed.3c00571 (PMC10938634; doi:10.1021/acs.jchemed.3c00571)
Supplement: Supplementary file 1 — ed3c00571_si_001.pdf [file ed3c00571_si_001.pdf]

Comparing Learning Outcomes, Student and Instructor Perceptions of a Simultaneous Online versus In-Person Biochemistry Laboratory Course

Laura Rowe

Department of Chemistry, Eastern Kentucky University, Richmond, KY, 40475, USA,
*laura.rowe@eku.edu

Supporting Information: Lab Report Sheets

Lab Report Sheet 1: Aseptic technique, prep work, pouring and streaking plates

Your Name (print)

1.) Write the work flow that we will be doing in this lab when purifying and identifying plasmid DNA. In addition to what is written on the board, each step should explain *why* we are doing those particular steps.

2.) You need to use aseptic technique when you are growing genetically engineered bacteria, but you don't need to use aseptic technique after your bacterial cells have fully grown and you are starting to purify your protein of interest. Why?

3.) What does LB in LB Broth Lennox stand for? What are the ingredients of LB Broth Lennox?

4.) What is the difference between agar (what we used today) and agarose (what we will use for our DNA gels)?

5.) What is the purpose of the 'triple Z-streak' we used when plating bacteria?

6.) We will be using plasmid DNA that codes for sf-GFP.

a.) What does the "sf" in sf-GFP stand for?

b.) What is the difference between sf-GFP and regular, or wild-type GFP? Cite your source.

c.) What is the excitation and emission fluorescence maximum of sf-GFP? Cite your source.

7.) The plasmid in our *E. coli* bacteria contains the gene for sf-GFP and a gene for resistance to the antibiotic ampicillin. What is the purpose of adding ampicillin to all of the food (broth/agar) that we grow our bacteria in?

8.) Is *Escherichia coli* a gram-negative or gram-positive bacteria? And what does 'gram-negative' or 'gram-positive' bacteria even mean?

Report Sheet 2: DNA Plasmid Purification, Restriction Enzyme, and Agarose Gel.

Your Name (print)

1.) Last week you purified your plasmid DNA using a Qiagen mini-prep kit. Use the following link to access the QIAPrep Miniprep Handbook in order to answer the following questions. You may use other internet sources as well, if needed. Please note the ethanol and RNAseA was added to your buffers prior to you using them, and remember that we used the microcentrifuge method and not the vacuum manifold method.

a.) In your own words, summarize how this kit successfully purified your plasmid DNA from the cell?

b.) Which buffer was used to lyse open the cells, and what ingredients caused cell lysis, and why?

c.) Which buffer caused the chromosomal DNA and denatured protein to precipitate? Why/how/what ingredient in the buffer caused this?

d.) After the 10 minute centrifugation step what was present in the precipitate? Why was your plasmid DNA not in the precipitate?

e.) The protocol says you can use EB buffer OR water to elute your plasmid through the filter in the final step. Why could you use either?

2.) On our agarose gel we will be migrating DNA that is between 500 bp and 7000 bp. Answer the following questions.

a.) If you needed to migrate and visualize DNA between 15,000 and 25,000 bp would you need an agarose gel with more or less agarose percentage? Why/why not?

b.) Could you/should you use the same molecular weight marker we used in class if you were migrating the DNA in a.? Why/why not?

3.) The Nanodrop instrument you used very conveniently calculates your DNA concentration for you. However, it does so using Beer's law and the fact that dsDNA absorbs 260 nm UV light. An A_{260} reading of 1.0 = 50 microg/mL pure dsDNA, and most instruments have a linear range between 0.1-1.0 A_{260}. If readings fall outside that linear range the absorbance measurement is not accurate and either more concentrated DNA or more diluted DNA must be used.

a.) If you were using a regular spectrophotometer to measure the UV absorbance of your DNA sample, and had a 1 cm cuvette (so a 1 cm pathlength), what concentration of DNA would you have if the instrument gave an $A_{260} = 0.21$? Show your work.

b.) Assume your sample in a. above was diluted from the original sample because the original sample gave a reading of 1.9 A_{260} absorbance, which was outside the linear range of the instrument. Below are the volumes that were used to make the dilution that was used in a. According to your concentration in a.) and your dilution factor, what was the original concentration of DNA in this sample, before dilution? Show your work.

100 microL of the original DNA sample was added to 900 microL of buffer, and mixed. This diluted sample was then used in the spectrophotometer and gave an $A_{260} = 0.21$.

4.) Use the plasmid map in your lab manual to answer the following questions.

a.) You digested your plasmid with HindIII and MluI. What size fragments should you see on your agarose gel IF your DNA completely digested? Show your work.

b.) What size fragments do you actually see on your gel? Attach an image of your gel to this lab report, indicating which lane is your lane and writing in which bands in the MW ladder represent what MW. Online lab: I will provide you with an image.

c.) If b is different than a, then explain this result and hypothesize as to what extra bands are/why you don't have the expected bands/etc.

d.) Your plasmid uses the PBAD promoter and the addition of L-arabinose to induce expression of the sf-GFP gene (L-arabinose must be present for the sf-GFP gene to be transcribed – so that the sf-GFP mRNA can then be translated to sf-GFP protein). Use the internet to look up how the pBAD promoter works and explain why/how (at a DNA level) your sf-GFP gene will be expressed/transcribed if there is L-arabinose in the growth media but will not be expressed/transcribed if there is no L-arabinose in the growth media. Do not exceed the space provided.

Lab Report Sheet 3: Protein Purification and SDS-PAGE

Your Name (print)

1.) Write the work flow that you used to express your protein, purify it, and check for purity using SDS-PAGE.

2.) Use your protein concentration data (from either the Nanodrop or microtiter plate reader) to prepare a graph on Excel in which Fraction # is on the x-axis and either Abs at 280 nm or protein concentration in mg/mL is on the y axis. Label your graph and axis as is typical, and print a copy and attach to this sheet. Use a pen/pencil to manually draw in the curve connecting the points on your graph.

3.) Use your fluorescence data from the microtiter plate reader to prepare a graph on Excel in which Fraction # is on the x-axis and fluorescence is on the y axis (RLU is the unit for fluorescence). When preparing this graph be sure to blank subtract your values – the blank values for different buffers are given for you in your screen shot data on BB. Label your graph and axis as is typical, and print a copy of your graph and attach to this sheet. Also attach a copy of your screen shot data from BB, indicating which fractions were yours. Use a pen/pencil to manually draw in the curve connecting the points on your graph.

4.) Attach an image of your SDS-PAGE gel, marking (with pen or Sharpee) on the image both where your protein fractions are (which ones are in which lane) and marking some of the MW ladder lanes with the correct MW according to your MW ladder key.

5.) According to your #2 graph, which fractions had the highest amount of protein? If these were not the same fractions that had the highest amount of fluorescence, explain why this was the case.

6.) According to your #3 graph, which fractions had the highest amount of sf-GFP? Did these correspond to elution buffer fractions? If yes, why would that be the case? If no, rationalize why that might be in your case.

7.) According to your SDS-PAGE results, do you have any fractions that show >90% pure sf-GFP? If you say yes, explain which ones and why you think so. If you say no, explain why you think that and suggest a ‘next experimental step’ to take in order to further purify your protein.

8.) Why do most proteins absorb light at 280 nm?

9.) It is true that a 6x His tags (and thus His amino acids) bind very well to immobilized Ni^{2+} in your column you used for protein purification. However, at physiological pH, what other R groups of amino acids would bind to an immobilized nickel column?

10.) What is the advantage of using high, or higher, pressure liquid chromatography systems for purifying protein – such as HPLC, UHPLC, and FPLC.

11.) Define the abbreviations HPLC, UHPLC, and FPLC, and explain why FPLC is usually used for protein purification instead of HPLC or UHPLC (although these are sometimes used as well).
