# Supplementary material for: Comparing Learning Outcomes and Student and Instructor Perceptions of a Simultaneous Online versus In-Person Biochemistry Laboratory Course
Source: J Chem Educ. 2024 Feb 5;101(3):882–91. doi: 10.1021/acs.jchemed.3c00571 (PMC10938634; doi:10.1021/acs.jchemed.3c00571)
Supplement: Supplementary file 3 — ed3c00571_si_003.pdf [file ed3c00571_si_003.pdf]

Comparing Learning Outcomes, Student and Instructor Perceptions of a Simultaneous Online versus In-Person Biochemistry Laboratory Course

Laura Rowe

Department of Chemistry, Eastern Kentucky University, Richmond, KY, 40475, USA, *laura.rowe@eku.edu

Supporting Information: Assessment Grading Rubrics for Scored Assessments

Lab Report Sheet 1: Aseptic technique, prep work, pouring and streaking plates.

SLO 1: Summarize procedural steps and state purpose of each step.

Procedural Steps and Purpose Lab Report 1

**Procedural Step**	**Purpose of Step**
Prepare LB agar	Food for bacteria that will form a gel-like solid on plates
Autoclave LB agar	Sterilize the food for bacteria
Add antibiotic to LB agar after slightly cooled	So only the desired (antibiotic resistant) bacteria will grow on plate, done after autoclaving because many antibiotics are heat-labile
Pour LB agar into plate and let cool	To have a gel-like food material for bacteria to grow on
Triple Z streak plate with glycerol stock of bacteria	To spread bacteria onto plate to grow, triple Z used to make sure colonies are spread out enough to be separated from on another
Incubate streaked plates overnight at 37 °C	Give bacteria time to grow
Pick colony off of incubated plate and incubate in liquid LB Broth (+ antibiotics) with shaking overnight at 37 °C	Grow more of one colony of bacteria to have enough for subsequent procedures

Rubric Scoring:

Each correct step = 1 point, each correct purpose = 1 step. 14 points possible

**Points Earned**	**Rubric Score**
0	1
1	2
2	3
3-4	4
5-6	5
7-8	6
9-10	7
11	8
12	9
13+	10

SLO 2: Understand purpose of procedural steps.

Question #	Question
2	You need to use aseptic technique when you are growing genetically engineered bacteria, but you don't need to use aseptic technique after you bacterial cells have fully grown and you are starting to purify your protein of interest. Why?
5	What is the purpose of the "triple Z-streak" we used when plating bacteria?
7	The plasmid in our E. coli bacteria contains the gene for sf-GFP and a gene for resistance to the antibiotic ampicillin. What is the purpose of adding ampicillin to all of the food that we grow our bacteria in?

Correct answers: 2-because you will be breaking open cells and getting protein out now, no longer growing bacteria, 5- getting bacterial broth dilute enough to have single colonies, 7-so only our desired bacteria grows.

Rubric Scoring:

Question not answered correctly at all = 0 pts, question answered partially correct = 1 pt, question answered correctly = 2 pts. 3 questions total = 6 possible points

Points Earned	Rubric Score
0	1
1	2
2	3
3	4
4	6
5	8
6	10

<u>SLO 3: Use resources provided and available online to gather, understand, and explain/state important information about laboratory procedures.</u>

Question #	Question
3	What does LB in LB Broth Lennox stand for? What are the ingredients of LB Broth Lennox?
4	What is the difference between agar and agarose?
6	We will be using plasmid DNA that codes for sf-GFP.<br>a.) What does the "sf" is sf-GFP stand for?<br>b.) What is the difference between sf-GFP and regular, or wild-type GFP?<br>c.) What is the excitation and emission fluorescence maximum of sf-GFP? Cite your source.
8	Is E. coli a gram-negative or a gram-positive bacteria? And what does "gram-negative" or "gram-positive" even mean?

Correct answers: 3- luria bertrani, see ingredients on label, 4-agar is used to solidify food plates and agarose is used for making DNA gels, 6-superfolder, it folds better, see literature they cited, 8-gram-negative, see relevant definitions.

Rubric Scoring:

Some of these questions had multiple parts, #3 had two parts, #6 had 4 different parts (a,b,c, and citing the source), and #8 had two different parts. Each of these parts counted as a different question in rubric scoring, for a total of 9 "sub-questions." Question or subquestion not answered correctly at all = 0 pts, question answered partially correct = 1 pt, question answered correctly = 2 pts. 9 questionsub-questions total = 18 possible points

Points Earned	Rubric Score
0	1
1-2	2
3-4	3
5-6	4
7-8	5
9-10	6
11-12	7
13-14	8
15-16	9
17-18	10

SLO 4: Analyze experimental results and determine "success" of procedures. Not applicable for Report Sheet 1.

<u>Lab Report Sheet 2: DNA Plasmid Purification, Restriction Enzyme, and Agarose Gel.</u>

<u>SLO 1: Summarize procedural steps and state purpose of each step. Not applicable for Lab Report Sheet 2</u>

<u>SLO 2: Understand purpose of procedural steps.</u>

**Question #**	**Question**
1a.	In your own words, summarize how this kit successfully purified your plasmid from the cell?
1d.	After the 10 minute centrifugation step what was present in the precipitate? Why was your plasmid DNA not in the precipitate?
1e.	The protocol says you can use EB buffer OR water to elute your plasmid through the fiter in the final step. Why could you use either?

The correct answers to 1d. and 1e. can be found in the Qiagen Mini-Prep Purification Manual. The correctness of 1a. will vary on actual results.

Rubric Scoring:

There are 2 parts to 1d., such that there are 4 questions/sub-questions total. Question not answered correctly at all = 0 pts, question answered partially correct = 1 pt, question answered correctly = 2 pts. 4 questions total = 8 possible points

**Points Earned**	**Rubric Score**
0	1
1	2
2	3
3	4
4	5-6
5	7
6	8
7	9
8	10

SLO 3: Use resources provided and available online to gather, understand, and explain/state important information about laboratory procedures.

Question #	Question
1b.	Which buffer was used to lyse open the cells, and what ingredients causes cell lysis, and why?
1c.	Which buffer caused the chromosomal DNA and denatured protein to precipitate? Why/how/what ingredient in the buffer caused this?
2a.	If you needed to migrate and visualize DNA between 15,000 and 25,000 bp would you need an agarose gel with more or less agarose percentage? Why/why not?
2b.	Could you/should you use the same molecular weight marker we used in class if you were migrating the DNA in a.? Why/why not?
4d	Your plasmid uses the pBAD promoter and the addition of L-arabinose to induce expression of the sf-GFP. Use the internet to look up how the pBAD promoter works and explain why/how (at a DNA level) your sf-GFP gene will be expressed/transcribed if there is L-arabinose in the growth media but will not be expressed/transcribed if there is no L-arabinose in the growth media.

The correct answers to 1b, 1c, and 2a. can be found in the Users Manual for the Qiagen Mini-Prep Kit. 2b answer varies depending on what DNA was used in the course.

Rubric Scoring:

Some of these questions had multiple parts, #1b , 1c, 2a, and 2b all had two parts. Each of these parts counted as a different question in rubric scoring, for a total of 9 "questions/sub-questions." Question or subquestion not answered correctly at all = 0 pts, question answered partially correct = 1 pt, question answered correctly = 2 pts. 9 question/sub-questions total = 18 possible points

Points Earned	Rubric Score
0	1
1-2	2
3-4	3
5-6	4
7-8	5
9-10	6
11-12	7
13-14	8
15-16	9
17-18	10

SLO 4: Analyze experimental results and determine “success” of procedures.

Question #	Question
4a.	You digested your plasmid with HindIII and MluI. What size fragments should you see on your agorase gel if your DNA completely digested? Show your work.
4b.	What size fragments do you actually see on your gel? Attach an image of your gel to this lab report , indicating which lane is your lane and writing in which bands in the MW ladder represent what MW.
4c.	If b is different than a, then explain this result and hypothesize as to what extra bands are/why you don’t have expected bands/etc

Correct answer for 4a. and 4b. will determine on exactly which expression plasmid you use to express the sf-GFP. 4b. and 4c. will vary on actual experimental results.

Rubric Scoring:

Question 4b had 4 parts (size fragment, attached image, lane marking, MW ladder marked), for a total of 6 questions/sub-questions. Question or sub-question not answered correctly at all = 0 pts, question answered partially correct = 1 pt, question answered correctly = 2 pts. 6 question/sub-questions total = 12 possible points

Points Earned	Rubric Score
0	1
1	2
2	4
3	6
4	8
5	9
6	10

Lab Report Sheet 3: Protein Purification and SDS-PAGE.

SLO 1: Summarize procedural steps and state purpose of each step (purpose not asked)

Procedural Steps and Purpose Lab Report 3

**Procedural Step**
Grew overnight culture of bacteria with LB Lennox Broth + arabinose and ampicillin
Checked optical density at 600 nm wavelength
Centrifuge culture and keep the pellet
Added buffer, DNase, lysozyme, $MgSO_4$, and lysozyme to cell pellet and resuspended cell pellet
Freeze-thaw cycles of resuspended cell pellet
Centrifuge solution and keep supernatant
Use gravity IMAC column-add equilibration buffer, then add supernatant, then add equilibration and elution buffer and collect fractions
Measure absorbance at 280 nm for each fraction collected with Nanodrop
Measure the fluorescence emission (xx nm) of each fraction when excited with xx nm light
Run SDS-PAGE of selected fractions: Multiple steps
Stain and image SDS-PAGE gel: Multiple steps

Rubric Scoring:

Each correct step = 1 point, each correct purpose = 1 step. 22 points possible

**Points Earned**	**Rubric Score**
0-1	1
2	2
3	3
4	4
5	5
6	6
7	7
8	8

9	9
10	10

SLO 2: Understand purpose of procedural steps. Not applicable for Lab Report 3.

SLO 3: Use resources provided and available online to gather, understand, and explain/state important information about laboratory procedures.

**Question #**	**Question**
8.	Why do most proteins absorb light at 280 nm?
9.	It is true that 6x-His tags bind very well to immobilized Ni^{2+} in your column you used for protein purification. However, at physiological pH, what other R groups of amino acids would bind to an immobilized nickel column?
10.	What is the advantage of using high, or higher, pressure liquid chromatography systems for purifying proteins, such as HPLC, UHPLC, and FPLC?
11.	Define the abbreviations HPLC, UHPLC, and FPLC, and explain why FPLC is usually used for porotein purification instead of HPLC or UHPLC (although these are sometimes used as well).

Correct answers: 8., because of aromatic rings, 9. Histidine groups, 10. Faster separation and better resolution, 11. High performance, ultra high performance, and fast protein liquid chromatography. Because better at separating larger molecules.

Rubric Scoring:

Question #11 had two parts, each part counting as a different question in rubric scoring, for a total of 5 "questions/sub-questions." Question or subquestion not answered correctly at all = 0 pts, question answered partially correct = 1 pt, question answered correctly = 2 pts. 5 question/sub-questions total = 10 possible points

**Points Earned**	**Rubric Score**
1	1
2	2
3	3
4	4
5	5
6	6
7	7
8	8
9	9
10	10

SLO 4: Analyze experimental results and determine "success" of procedures.

Question #	Question
2.	Use your protein concentration data to create a graph of fraction # versus absorbance. (paraphrased)
3.	Use your fluorescence data to create a graph of fraction # versus fluorescence signal. (paraphrased)
4.	Attach an annotated image of your SDS-PAGE gel (paraphrased)
5.	According to your #2 graph, which fractions had the highest amount of protein? If these were not the same fractions that had the highest amount of fluorescence, explain why.
6.	According to your #3 graph, which fractions had the highest amount of sf-GFP? Did these correspond to you elution buffer fractions? If yes, why would that be the case? If no, rationalize why that might not be the case?
7.	According to your SDS-PAGE results, do you have any fractions that show >90% purity sf-GFP? If you say yes, explain which ones and why you think so. If you say no, explain why you think that and suggest a "next experimental step to take in order to further purify your protein.

Correct answers vary on results.

Rubric Scoring:

Question 5 had 2 parts, and questions 6 and 7 each had 2 parts, for a total of 9 questions/sub-questions. Question or sub-question not answered correctly at all = 0 pts, question answered partially correct = 1 pt, question answered correctly = 2 pts. 6 question/sub-questions total = 18 possible points

Points Earned	Rubric Score
0	1
1-2	2
3-4	3
5-6	4
7-8	5
9-10	6
11-12	7
13-14	8
15-16	9
17+	10

Final Lab Report: Journal Paper in JACS Format

SLO 1: Summarize procedural steps and state purpose of each step.

Procedural Steps and Purpose Final Lab Report

Rubric Scoring: Experimental/Materials and Methods section of article was assessed and scored according to the following rubric.

**Section Qualities**	**Rubric Score**
No Experimental section provided	1
Experimental section extremely brief and incomplete	2-3
Experimental section mentioned and described between 40-60% of procedural steps	4-6
Experimental section mentioned and described between 70-80% of procedural steps	7-8
Experimental section mentioned and described almost all procedural steps	9
Experimental section mentioned and described all procedural steps	10

SLO 2: Understand purpose of procedural steps.

Rubric Scoring: Results and Discussion sections of articles were assessed for descriptive mentions of what the purpose of the procedural step was.

**Section Qualities**	**Rubric Score**
No mention of purpose of any procedural steps	1
Purpose of between 10-50% or procedural steps discussed	2-5
Purpose of between 60-80% or procedural steps discussed	6-9
Purpose of between 90%+ or procedural steps discussed	10

SLO 3: Use resources provided and available online to gather, understand, and explain/state important information about laboratory procedures: Not applicable for Final Lab Report

<u>SLO 4: Analyze experimental results and determine "success" of procedures.</u>

Rubric Scoring: Results and Discussion sections of articles were assessed for descriptive mentions what their experimental results were and whether or not their experiment was successful.

**Section Qualities**	**Rubric Score**
No summary or interpretation of results	1
Minimal summary of results, but no interpretation.	2-5
Minimal summary of results, and minimal interpretation of results.	6-7
Average to good summary and interpretation of results	8-9
Good to excellent summary and interpretation of results	10

<u>SLO 5: Be able to read, interpret, and cite literature relevant to the semester laboratory project.</u>

Rubric Scoring: Entire final lab report was assessed for the following: Discussion of previous research, citing that research within the paper, correct reference format at end of paper, reasonable number of references cited, some primary references cited.

**Paper Qualities**	**Rubric Score**
No references cited and no previous work discussed.	0
Minimal references/citations/discussion of previous work.	2-5
Acceptable references/citation/discussion of previous work.	6-7
Good references/citation/discussion of previous work.	8-9
Excellent references/citation/discussion of previous work.	10

Learning Goal 1: Understand the entire semester's project as a whole research project, specifically how each week's experiments and results/data were necessary and informed the subsequent steps of the research project.

Paper Qualities	Rubric Score
Paper did not demonstrate significant understanding of sequence of procedural steps, and/or what the purpose of the experiments were and/or how to assess and interpret their data.	1-3
Paper demonstrated minimal understanding of sequence of procedural steps, and/or what the purpose of the experiments were and/or how to assess and interpret their data.	3-5
Paper demonstrated acceptable understanding of sequence of procedural steps, and/or what the purpose of the experiments were and/or how to assess and interpret their data.	6-7
Paper demonstrated good understanding of sequence of procedural steps, and/or what the purpose of the experiments were and/or how to assess and interpret their data.	8-9
Paper demonstrated excellent understanding of sequence of procedural steps, and/or what the purpose of the experiments were and/or how to assess and interpret their data.	10

Learning Goal 2: Successfully present and interpret the experimental results of the semester in a scientific journal format.

Paper Qualities	Rubric Score
Paper was missing entire sections, did not have any figures or data analysis, and/or was written with very poor writing style, formatting, and grammar.	1-3
Paper had all, or most, required sections, had at least one figure, and/or minimal data analysis, and/or poor writing style, formatting, and grammar.	3-5
Paper had all required sections, and had acceptable figures, and/orl data analysis, and/or writing style, formatting, and grammar.	6-7
Paper had all required sections, and had good figures, and/orl data analysis, and/or writing style, formatting, and grammar.Good references/citation/discussion of previous work.	8-9
Paper had all required sections, and had excellent figures, and/orl data analysis, and/or writing style, formatting, and grammar.	10
