# Supplementary material for: Comparing Learning Outcomes and Student and Instructor Perceptions of a Simultaneous Online versus In-Person Biochemistry Laboratory Course
Source: J Chem Educ. 2024 Feb 5;101(3):882–91. doi: 10.1021/acs.jchemed.3c00571 (PMC10938634; doi:10.1021/acs.jchemed.3c00571)
Supplement: Supplementary file 5 — ed3c00571_si_005.pdf [file ed3c00571_si_005.pdf]

Comparing Learning Outcomes, Student and Instructor Perceptions of a Simultaneous Online versus In-Person Biochemistry Laboratory Course

Laura Rowe

Department of Chemistry, Eastern Kentucky University, Richmond, KY, 40475, USA, *laura.rowe@eku.edu

Histograms of Student Learning Objectives and Learning Goals Scores from Assessments

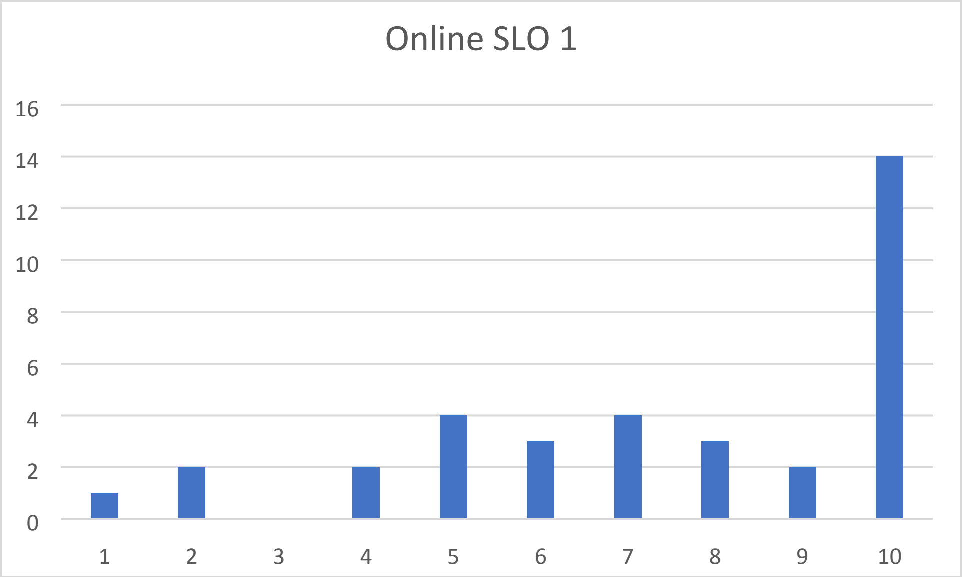

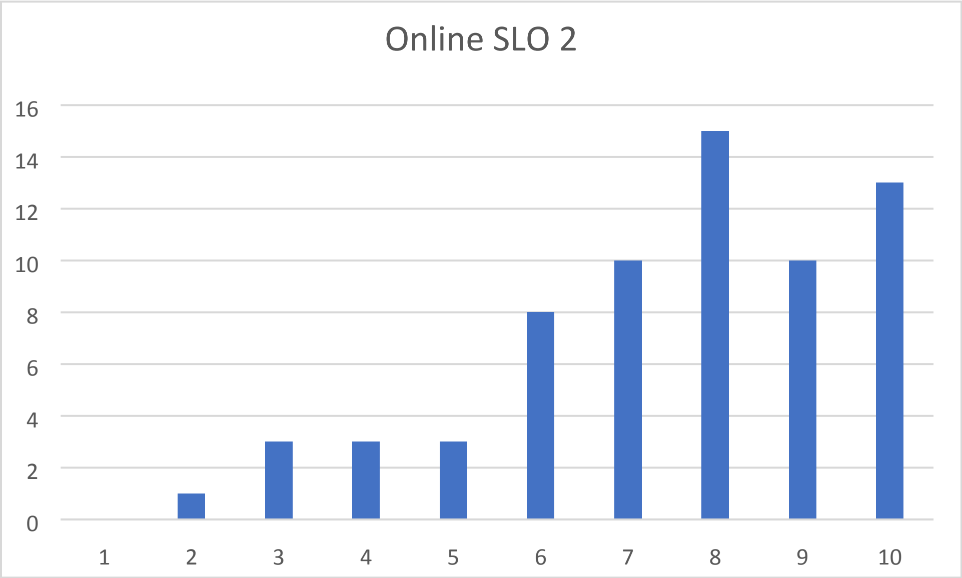

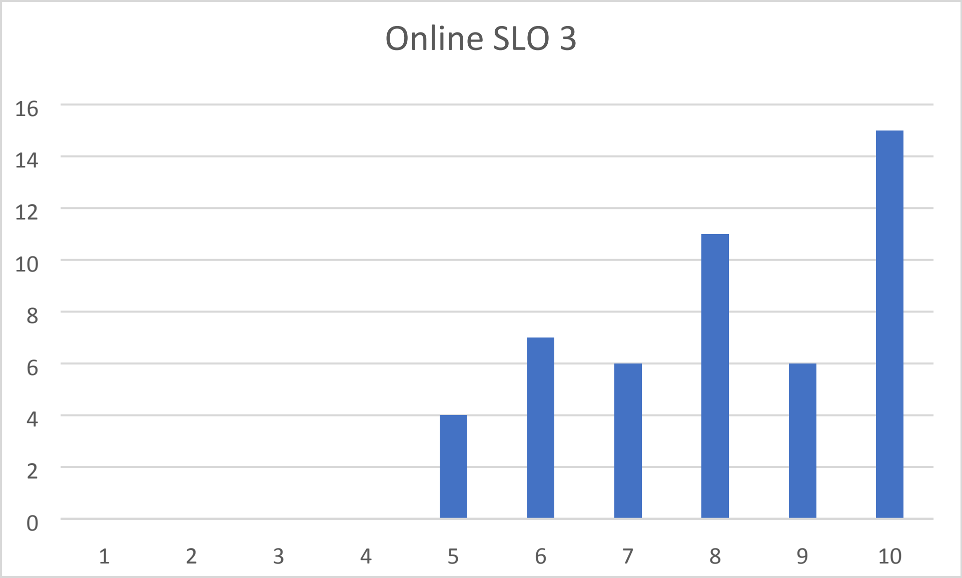

Online SLO 3
16
14
12
10
8
6
4
2
0
1
2
3
4
5
6
7
8
9
10

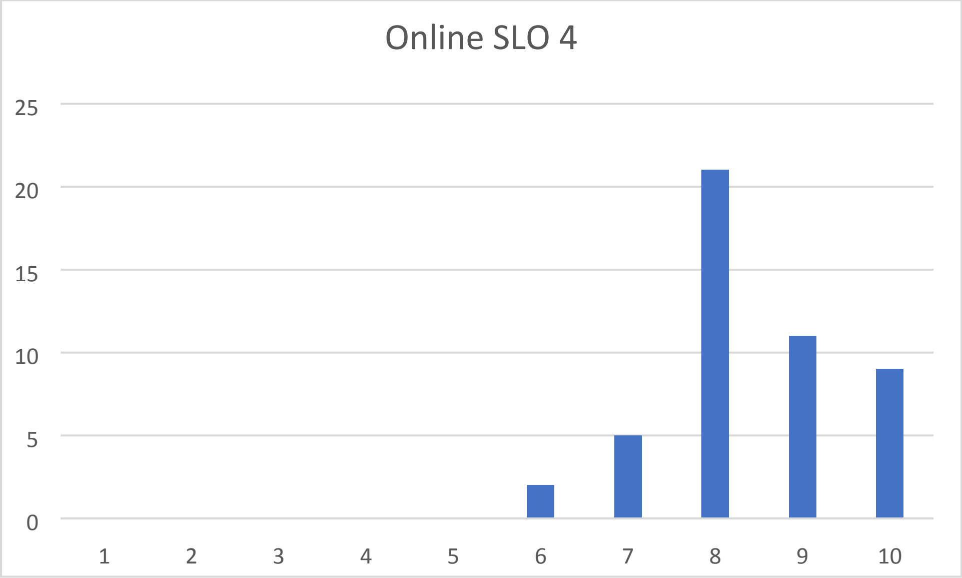

Online SLO 4
25
20
15
10
5
0
1
2
3
4
5
6
7
8
9
10

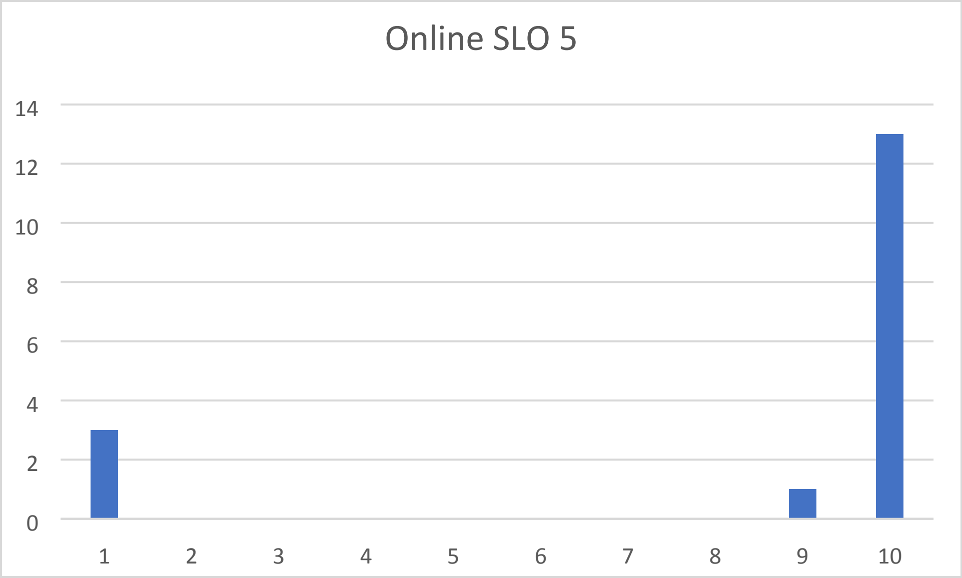
Online SLO 5
14
12
10
8
6
4
2
0
1
2
3
4
5
6
7
8
9
10

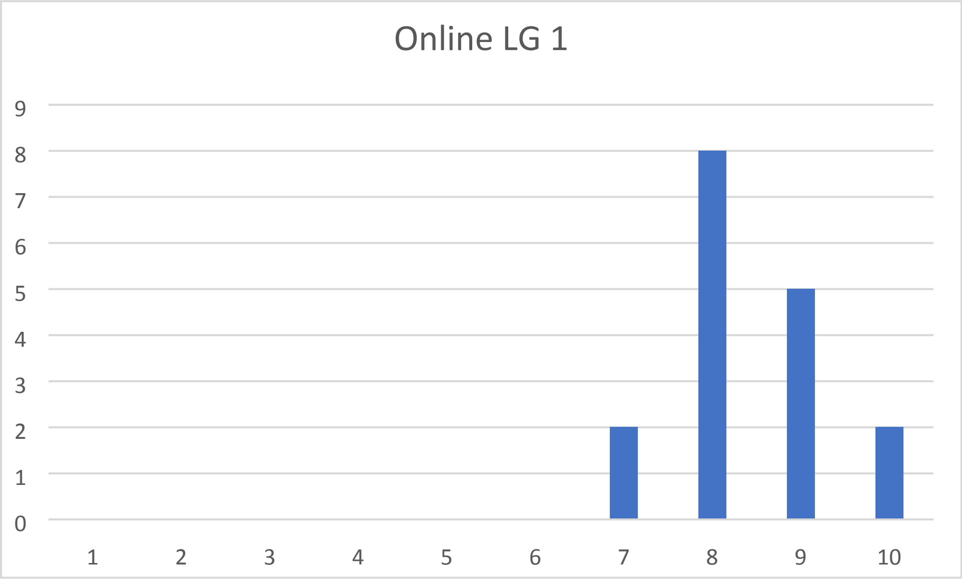
Online LG 1
9
8
7
6
5
4
3
2
1
0
1
2
3
4
5
6
7
8
9
10

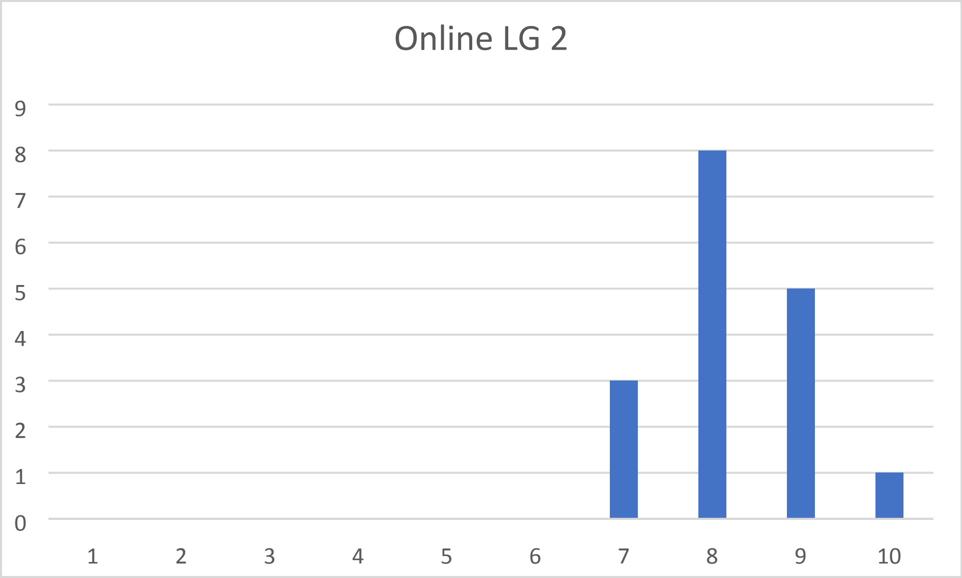
Online LG 2
0
1
2
3
4
5
6
7
8
9
1
2
3
4
5
6
7
8
9
10

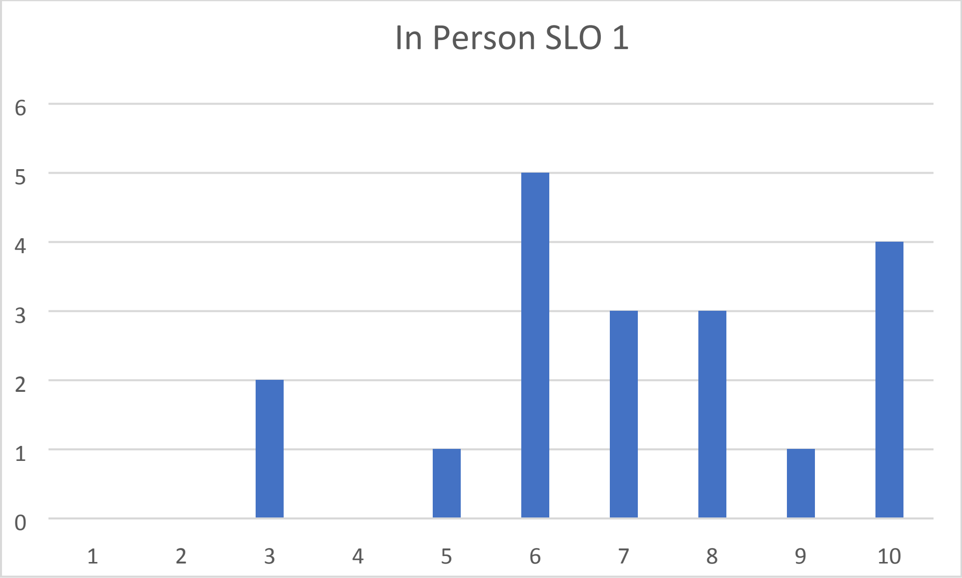
In Person SLO 1
0
1
2
3
4
5
6
1
2
3
4
5
6
7
8
9
10

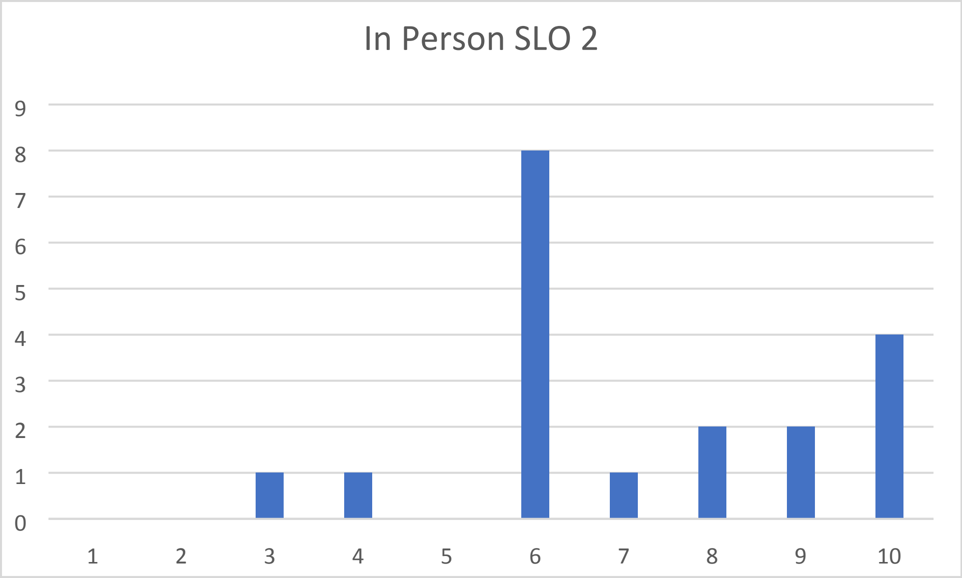
In Person SLO 2
0
1
2
3
4
5
6
7
8
9
1
2
3
4
5
6
7
8
9
10

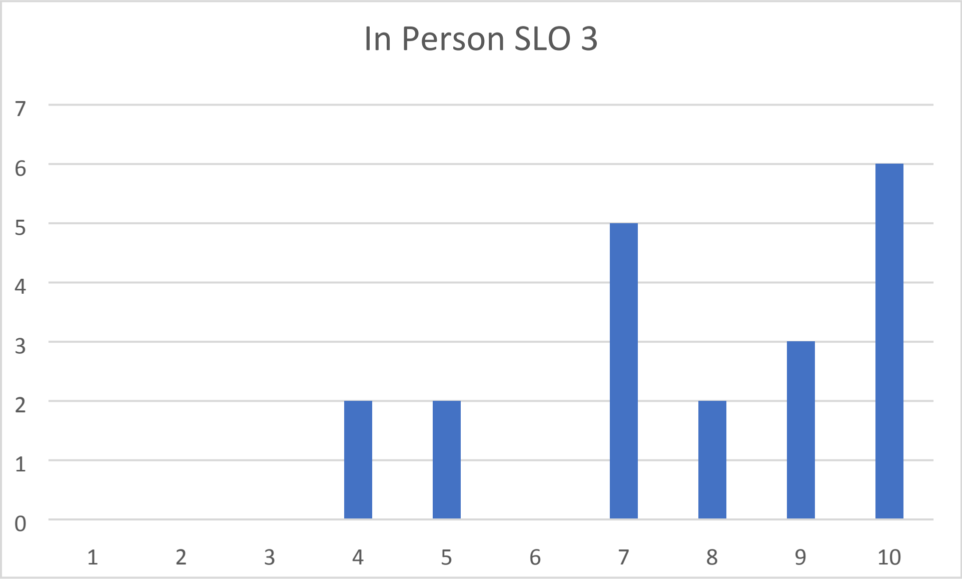
In Person SLO 3
0
1
2
3
4
5
6
7
1
2
3
4
5
6
7
8
9
10

Supporting Information

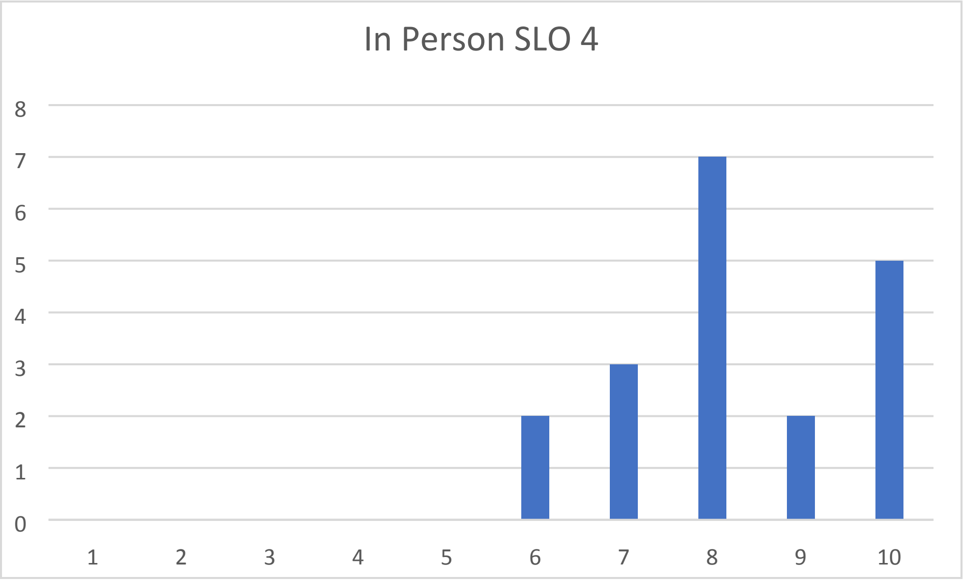

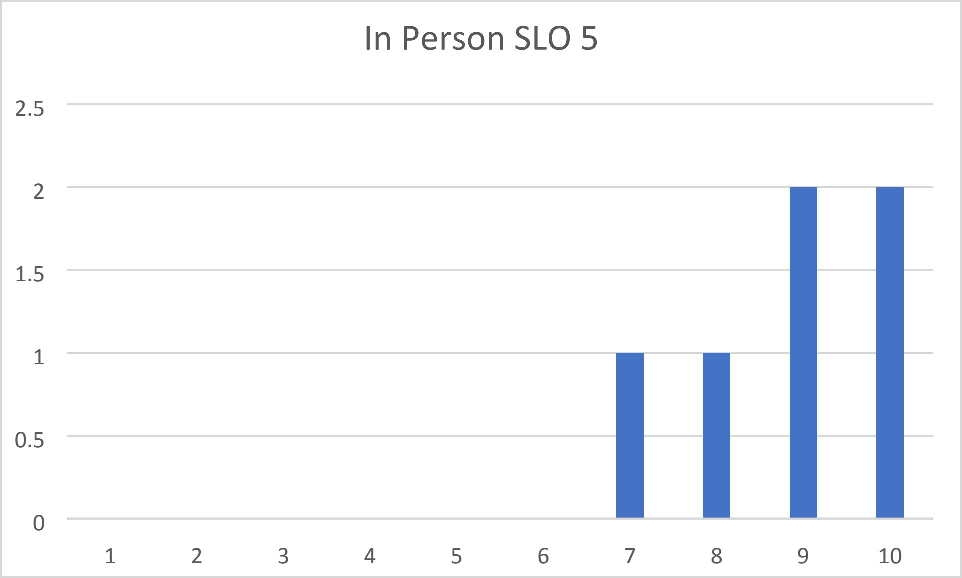
In Person SLO 5
2.5
2
1.5
1
0.5
0
1 2 3 4 5 6 7 8 9 10

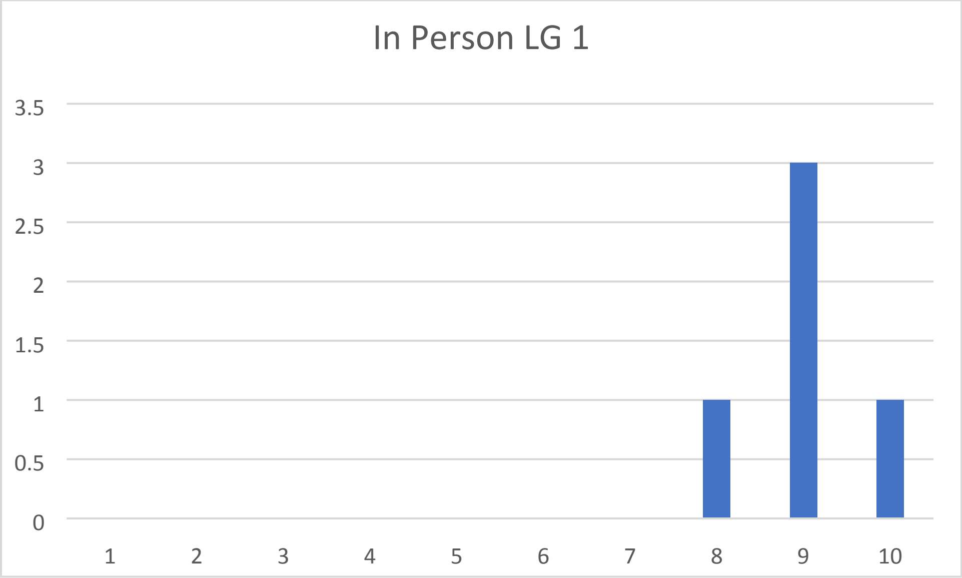
In Person LG 1
3.5
3
2.5
2
1.5
1
0.5
0
1 2 3 4 5 6 7 8 9 10

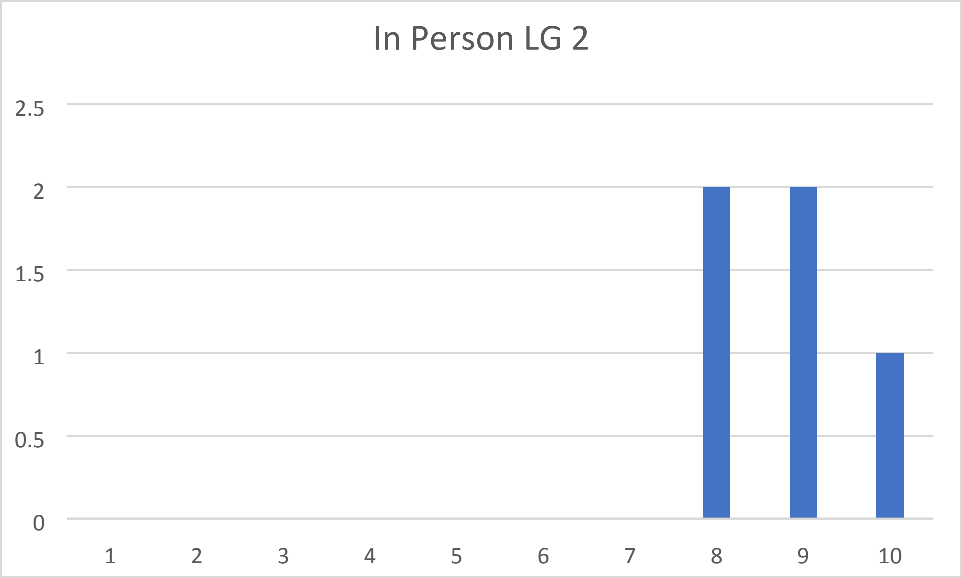
In Person LG 2
2.5
2
1.5
1
0.5
0
1
2
3
4
5
6
7
8
9
10
