# Supplementary material for: Comparing Learning Outcomes and Student and Instructor Perceptions of a Simultaneous Online versus In-Person Biochemistry Laboratory Course
Source: J Chem Educ. 2024 Feb 5;101(3):882–91. doi: 10.1021/acs.jchemed.3c00571 (PMC10938634; doi:10.1021/acs.jchemed.3c00571)
Supplement: Supplementary file 6 — ed3c00571_si_006.docx [file ed3c00571_si_006.docx]

Comparing Learning Outcomes, Student and Instructor Perceptions of a Simultaneous Online versus In-Person Biochemistry Laboratory Course

Laura Rowe

Department of Chemistry, Eastern Kentucky University, Richmond, KY, 40475, USA, [*laura.rowe@eku.edu](mailto:*laura.rowe@eku.edu)

Histograms of Student Learning Objectives and Learning Goals Scores from Assessments
