# Supplementary material for: Comparing Learning Outcomes and Student and Instructor Perceptions of a Simultaneous Online versus In-Person Biochemistry Laboratory Course
Source: J Chem Educ. 2024 Feb 5;101(3):882–91. doi: 10.1021/acs.jchemed.3c00571 (PMC10938634; doi:10.1021/acs.jchemed.3c00571)
Supplement: Supplementary file 8 — ed3c00571_si_008.pdf [file ed3c00571_si_008.pdf]

Comparing Learning Outcomes, Student and Instructor Perceptions of a Simultaneous Online versus In-Person Biochemistry Laboratory Course

Laura Rowe

Department of Chemistry, Eastern Kentucky University, Richmond, KY, 40475, USA,
*laura.rowe@eku.edu

Anonymous Questionnaire About Online vs. In-Person Laboratory-Biochemistry

Background Questions:

1.) What is your major?

2.) What are your plans after college (science job, other job, medical school, graduate school, professional school, unsure, etc.)

3.) Estimate your current science GPA, and your current overall GPA:

4.) Did you take the in-person lab or the online lab for Biochem?

5.) Did you **want** to take the lab section/type you did, or did you have to due to scheduling reasons/other reasons?

6.) If you chose to take the online section of the lab, what was the top 1 or 2 reasons why you chose to take the online lab section. ***Any*** answer is acceptable, but some example reasons may be: thought it would be easier than in-person, didn't want to get up so early (in-person started at 8 am), don't like labs, thought it would take less time, didn't want to spend that much time in-person due to COVID concerns, etc.

Laboratory Experiences Reflection Questions:

For your experiences in either the online or in-person lab, please rank the following statements.

1.) **The lab section helped me understand the concepts taught in the lecture section.**

Strongly disagree Disagree Neither Agree or Disagree Agree Strongly Agree

2.) **I understood what was going on during the lab (the purpose of the lab, the reason certain procedures were being done, etc.).**

Strongly disagree Disagree Neither Agree or Disagree Agree Strongly Agree

3.) **I felt engaged completing the laboratory work/assignment (means to be interested and invested in understanding the information)**

Strongly disagree Disagree Neither Agree or Disagree Agree Strongly Agree

4.) **The lab portion of this course helped me prepare better for my future career**

Strongly disagree Disagree Neither Agree or Disagree Agree Strongly Agree

5.) **Completing the lab section of this course was useful.**

Strongly disagree Disagree Neither Agree or Disagree Agree Strongly Agree

6.) **My lab section was easier than I expected it to be.**

Strongly disagree Disagree Neither Agree or Disagree Agree Strongly Agree

7.) **My lab section was harder/more difficult than I expected it to be**

Strongly disagree Disagree Neither Agree or Disagree Agree Strongly Agree

8.) **This lab was comparable in ease/difficulty to other chemistry labs I have taken so far. If you disagree, please write in whether you found it easier or more difficult.**

Strongly disagree Disagree Neither Agree or Disagree Agree Strongly Agree

9.) **This lab was at a reasonable level of ease/difficulty for this course.**

Strongly disagree Disagree Neither Agree or Disagree Agree Strongly Agree

10.) **If I had to choose again, I would choose the section I chose for lab (whether you took online or in-person)**

Strongly disagree Disagree Neither Agree or Disagree Agree Strongly Agree

SA = strongly agree; A= agree, NAD = neither agree of disagree is NAD; D= disagree; SD = strongly disagree

Table 1. Did lab section help meet these potential goals/objectives of taking the lab?

	Statement					
1	To earn an A or B in the course	SA	A	NAD	D	SD
2	To prepare for the career I want to pursue	SA	A	NAD	D	SD
3	To develop my scientific writing skills	SA	A	NAD	D	SD
4	To make connections between lab and the real world	SA	A	NAD	D	SD
5	To understand how a chemistry research lab works	SA	A	NAD	D	SD
6	To learn lab techniques	SA	A	NAD	D	SD
7	To be efficient in lab	SA	A	NAD	D	SD
8	To prepare for future science courses	SA	A	NAD	D	SD
9	To connect concepts learned in lectures with laboratories	SA	A	NAD	D	SD
10	To work as a team	SA	A	NAD	D	SD
11	To learn how to design and carry out experiments	SA	A	NAD	D	SD
12	To carry out experiments safely	SA	A	NAD	D	SD
13	To apply lab techniques	SA	A	NAD	D	SD

Please list by number which of the goals/objectives in Table 1 were some of your personal goals/objectives in completing the lab section? For example, if the only objective you had for taking the lab section is because you had to in order to get an A or B in the course, just write in 1, etc.

Online Lab Students Only:

1.) **There were often multiple changes in what we actually did in lab (in videos), and what was initially in your lab manual. During the second half of the semester, your instructor highlighted the differences either in the "overview" video, or later, in a typed out document. Which method was more effective for you, to highlight changes from your lab manual? Circle one.**

Changes mentioned in overview video

Changes typed up in written document

Both were about the same in helpfulness

Neither were helpful

2.) **Which was more helpful to you to complete your lab notebook? Circle one:**

The posted videos

The information written in the lab manual

They were about the same in helpfulness

3.) **Which was more helpful to you to complete your lab sheets? Circle one:**

The posted videos

The information written in the lab manual

They were about the same in helpfulness

Additional Comments/Suggestions: Please write in any constructive criticism/suggestions for improvement here:
