# Supplementary material for: Comparing Learning Outcomes and Student and Instructor Perceptions of a Simultaneous Online versus In-Person Biochemistry Laboratory Course
Source: J Chem Educ. 2024 Feb 5;101(3):882–91. doi: 10.1021/acs.jchemed.3c00571 (PMC10938634; doi:10.1021/acs.jchemed.3c00571)
Supplement: Supplementary file 11 — ed3c00571_si_011.pdf [file ed3c00571_si_011.pdf]

Online Biochemistry Laboratory Perceptions Survey

Saturday, May 21, 2022

Powered by SurveyMonkey®

19

Total Responses

Date Created: Monday, January 10, 2022

Complete Responses: 19

Powered by SurveyMonkey

Q1: Was the online biochemistry lab you taught fully online, and NOT a hybrid laboratory in which students had a mixture of online and in-person labs.

Answered: 19 Skipped: 0

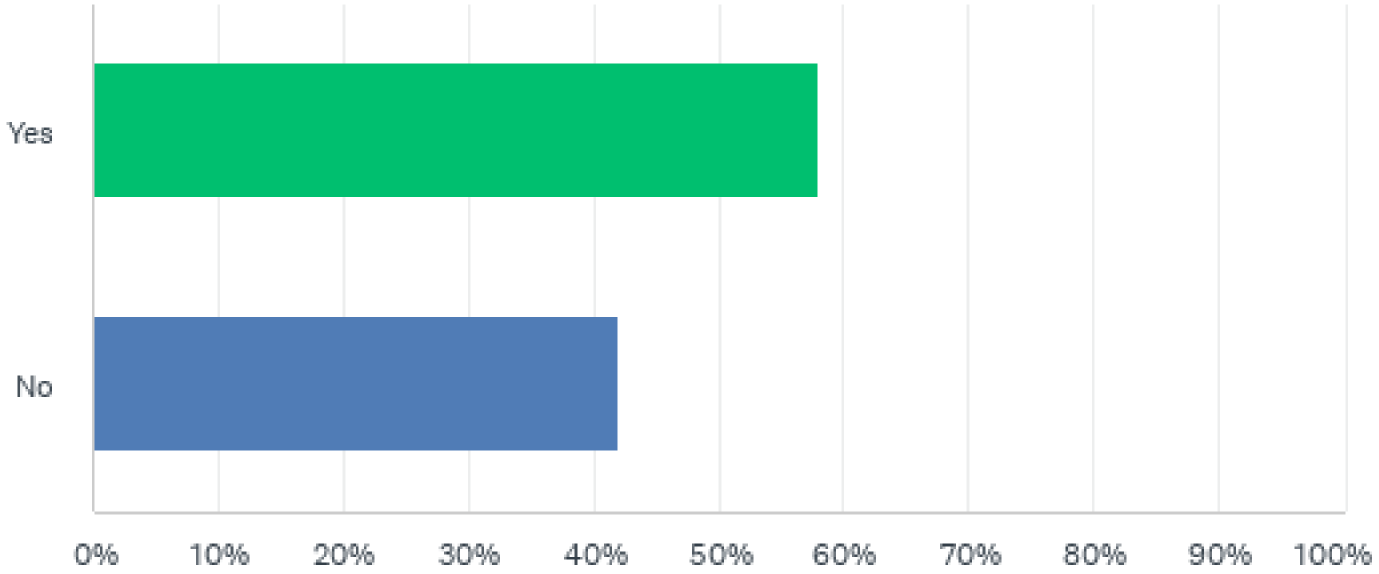

Powered by SurveyMonkey

Q1: Was the online biochemistry lab you taught fully online, and NOT a hybrid laboratory in which students had a mixture of online and in-person labs.

ANSWER CHOICES	RESPONSES	
Yes	57.89%	11
No	42.11%	8
TOTAL		19

Answered: 19   Skipped: 0

Powered by SurveyMonkey

Q2: Before the COVID-19 disruptions, had you ever taught an online biochemistry laboratory?

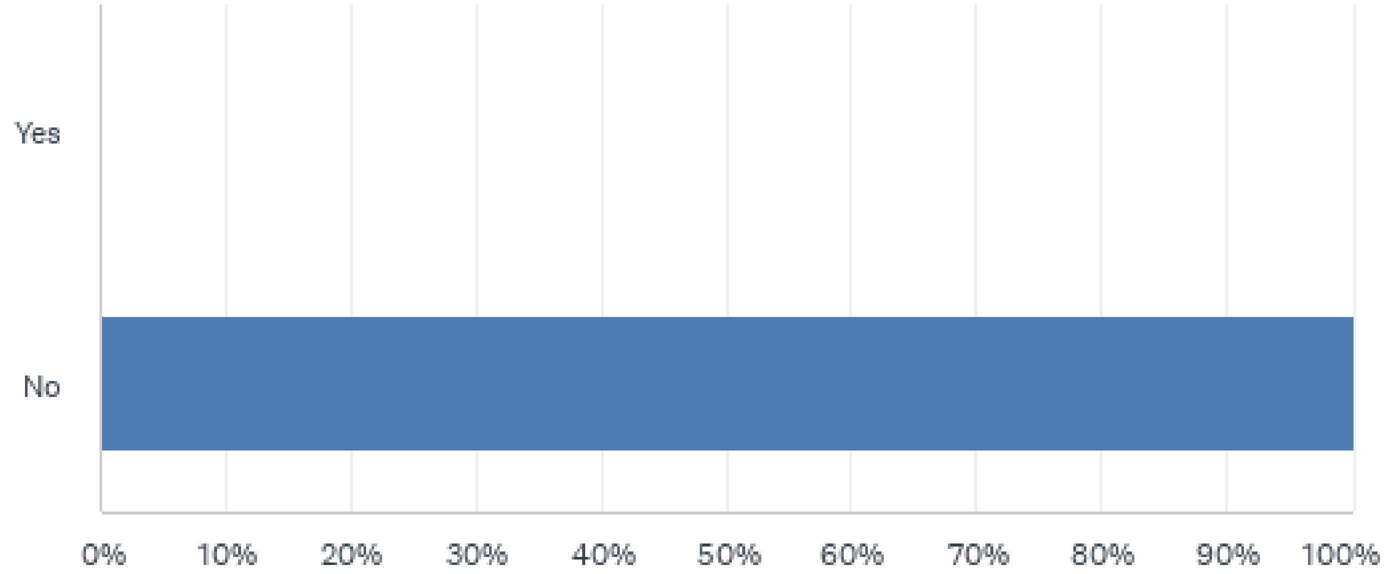

Answered: 19 Skipped: 0

Q2: Before the COVID-19 disruptions, had you ever taught an online biochemistry laboratory?

ANSWER CHOICES	RESPONSES	
Yes	0.00%	0
No	100.00%	19
TOTAL		19

Answered: 19 Skipped: 0

Powered by SurveyMonkey

Q3: Were you the instructor who taught the online biochemistry laboratory, or did a teaching assistant do all/most of the teaching?

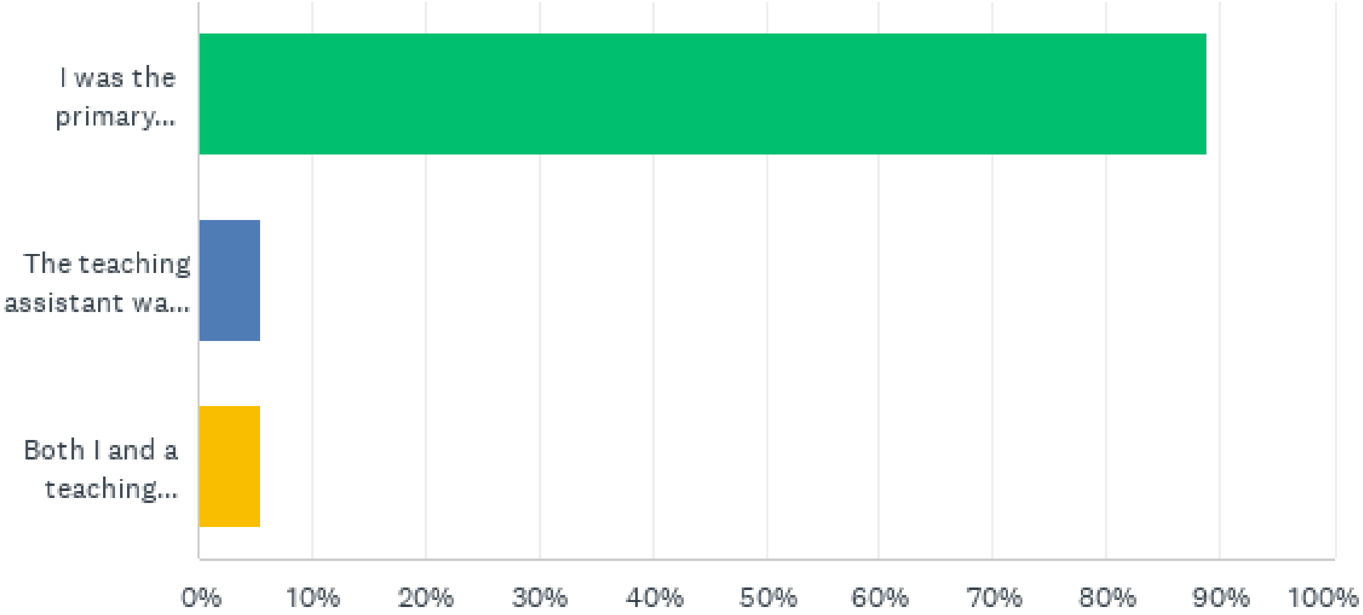

Answered: 18 Skipped: 1

Q3: Were you the instructor who taught the online biochemistry laboratory, or did a teaching assistant do all/most of the teaching?

Answered: 18   Skipped: 1

ANSWER CHOICES	RESPONSES	
I was the primary instructor for the lab.	88.89%	16
The teaching assistant was the primary instructor for the lab.	5.56%	1
Both I and a teaching assistant taught the lab.	5.56%	1
TOTAL		18

Q4: What was the class standing of most students who took the online laboratory class

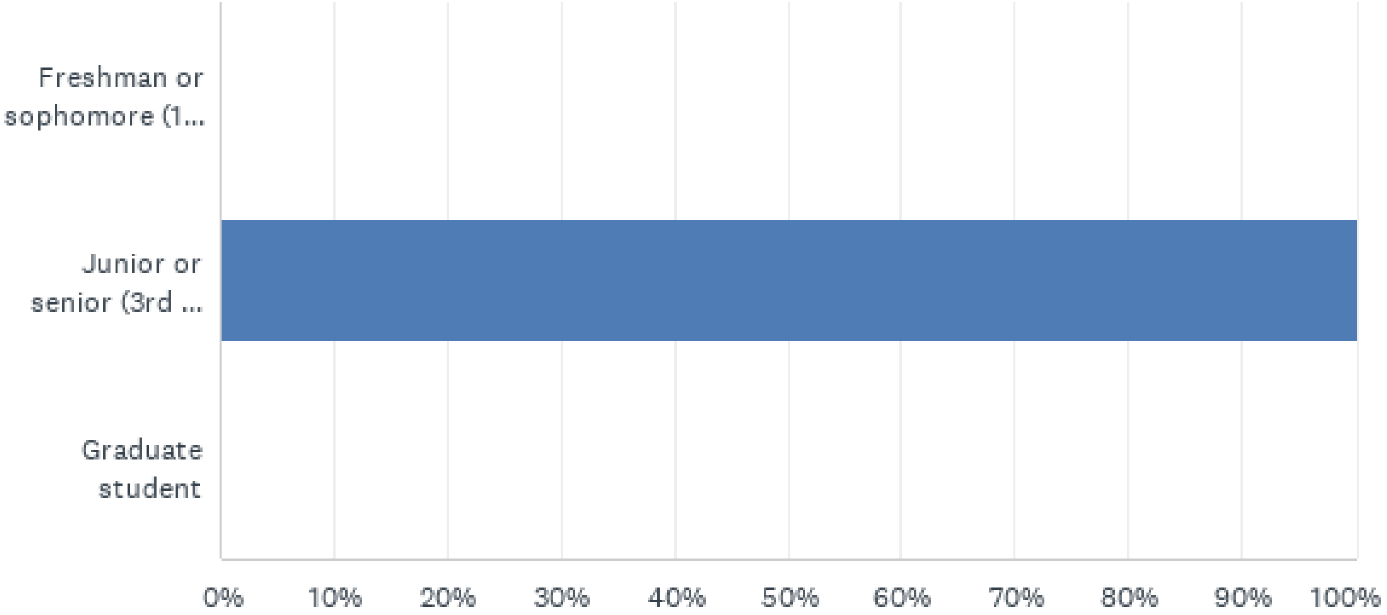

Answered: 19 Skipped: 0

Powered by SurveyMonkey

Q4: What was the class standing of most students who took the online laboratory class

ANSWER CHOICES	RESPONSES	
Freshman or sophomore (1st or 2nd year undergraduate)	0.00%	0
Junior or senior (3rd or 4th year undergraduate)	100.00%	19
Graduate student	0.00%	0
TOTAL		19

Answered: 19   Skipped: 0

Q5: Approximately how many students were in a section of your online biochemistry laboratory?

Answered: 19   Skipped: 0

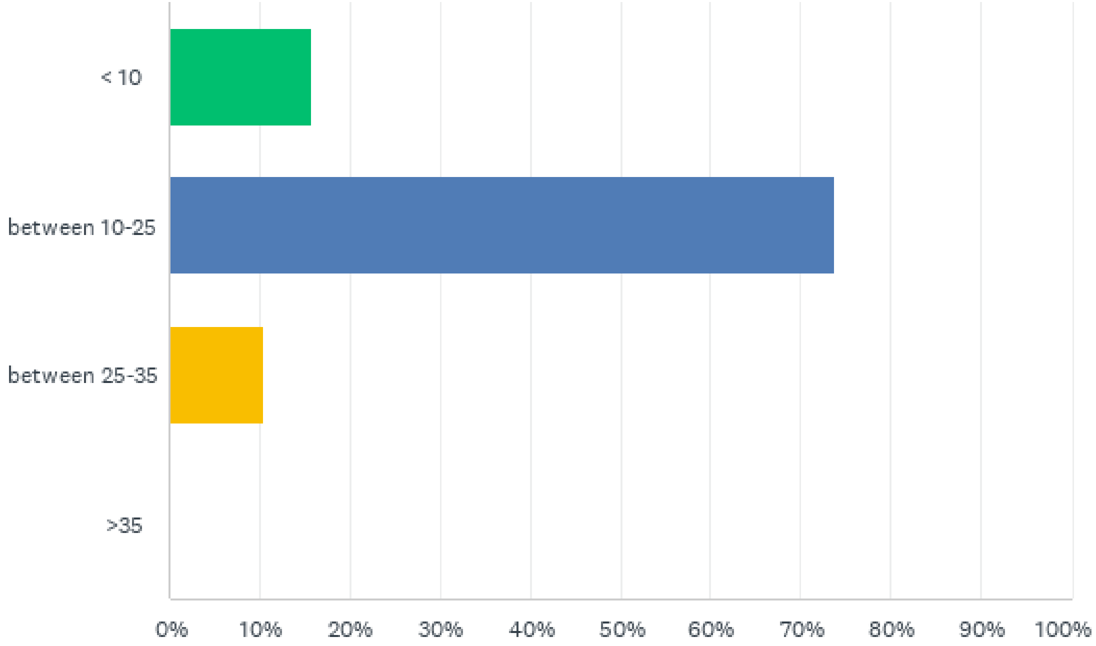

Powered by SurveyMonkey

Q5: Approximately how many students were in a section of your online biochemistry laboratory?

Answered: 19   Skipped: 0

ANSWER CHOICES	RESPONSES	
< 10	15.79%	3
between 10-25	73.68%	14
between 25-35	10.53%	2
>35	0.00%	0
TOTAL		19

Powered by SurveyMonkey

Q6: What modality was your online biochemistry laboratory?

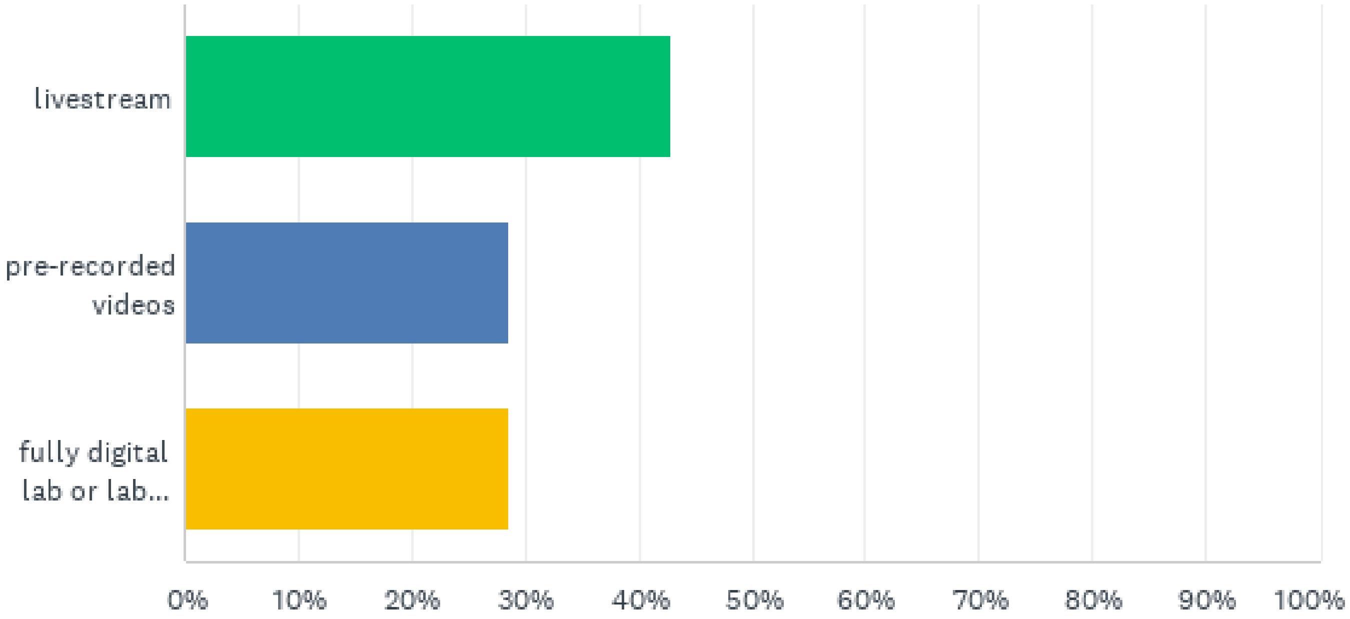

Answered: 14 Skipped: 5

Q6: What modality was your online biochemistry laboratory?

Answered: 14   Skipped: 5

ANSWER CHOICES	RESPONSES	
livestream	42.86%	6
pre-recorded videos	28.57%	4
fully digital lab or lab simulation	28.57%	4
TOTAL		14

Powered by SurveyMonkey

Q7: My institution is:

Answered: 19   Skipped: 0

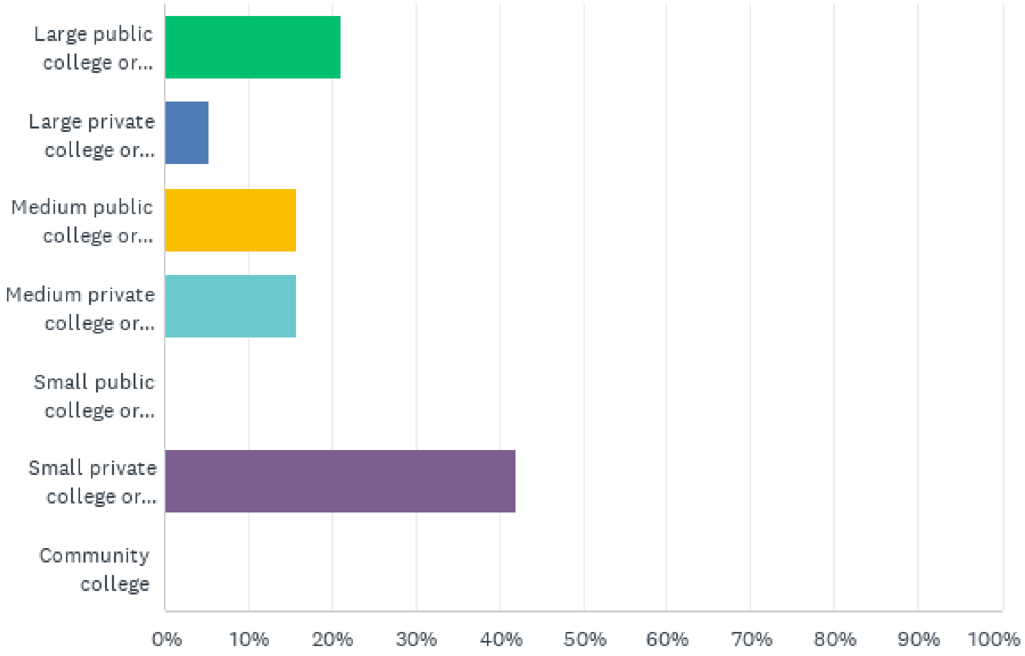

Q7: My institution is:

Answered: 19   Skipped: 0

ANSWER CHOICES	RESPONSES	
Large public college or university (>15,000 students)	21.05%	4
Large private college or university (>15,000 students)	5.26%	1
Medium public college or university (5000-15,000 students)	15.79%	3
Medium private college or university (5000-15,000 students)	15.79%	3
Small public college or university (<5000 students)	0.00%	0
Small private college or university (<5000 students)	42.11%	8
Community college	0.00%	0
TOTAL		19

Q8: The online only biochemistry laboratory was equivalent to an in-person biochemistry laboratory in terms of student learning.

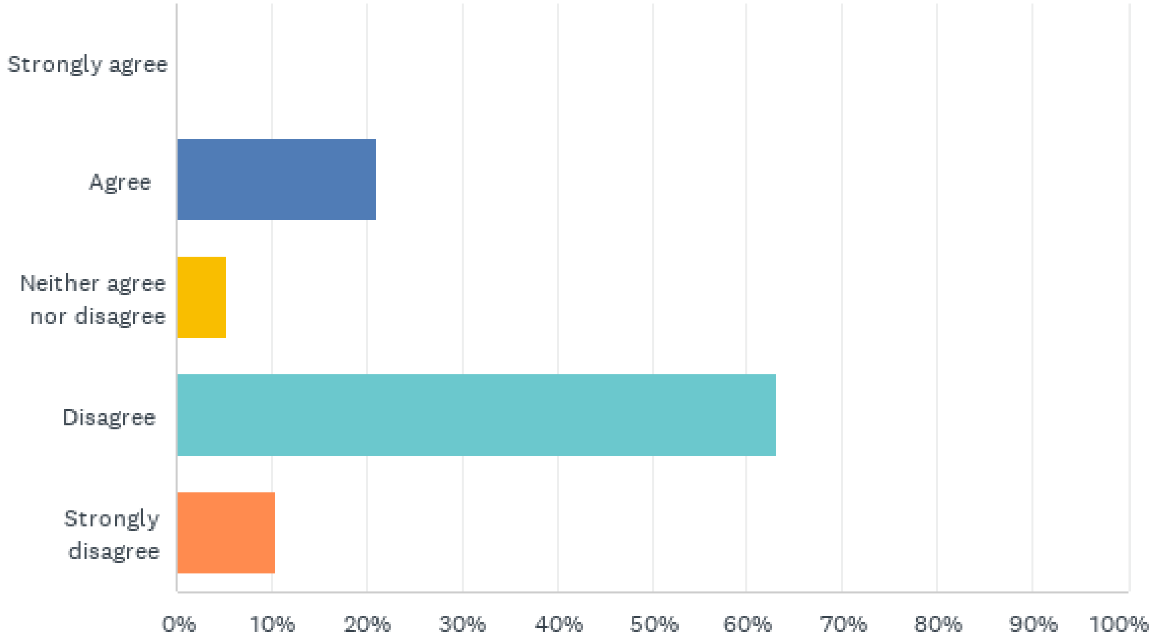

Answered: 19   Skipped: 0

Powered by SurveyMonkey

Q8: The online only biochemistry laboratory was equivalent to an in-person biochemistry laboratory in terms of student learning.

Answered: 19 Skipped: 0

ANSWER CHOICES	RESPONSES	
Strongly agree	0.00%	0
Agree	21.05%	4
Neither agree nor disagree	5.26%	1
Disagree	63.16%	12
Strongly disagree	10.53%	2
TOTAL		19

Powered by SurveyMonkey

Q9: The online only biochemistry laboratory required LESS time and effort from me, the instructor, than an in person biochemistry laboratory.

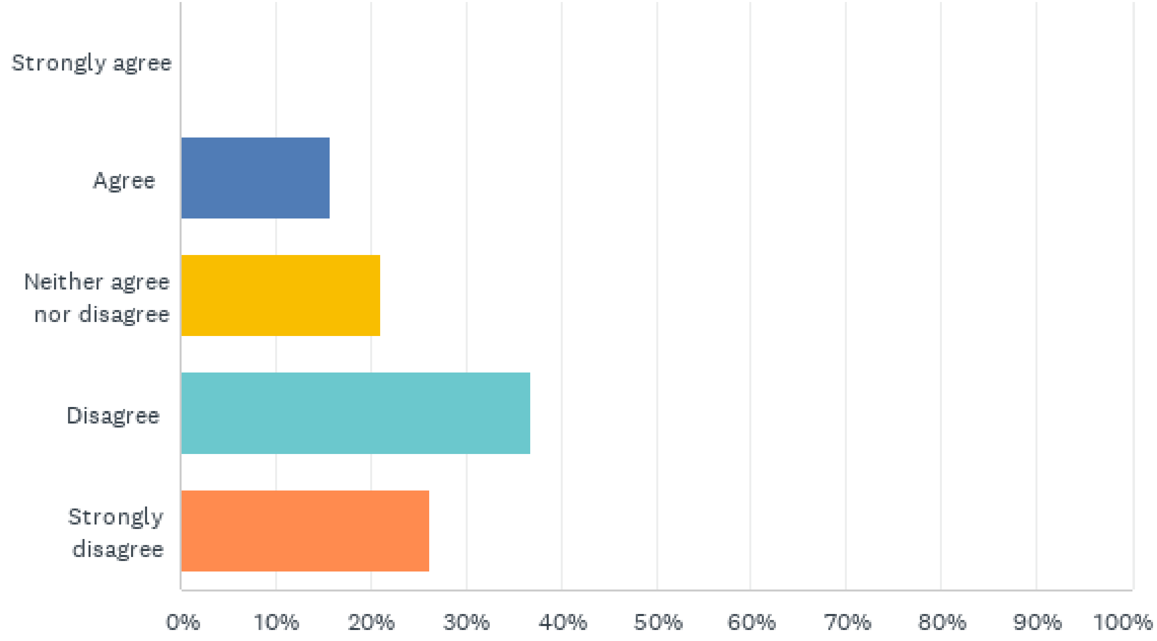

Answered: 19   Skipped: 0

Q9: The online only biochemistry laboratory required LESS time and effort from me, the instructor, than an in person biochemistry laboratory.

ANSWER CHOICES	RESPONSES	
Strongly agree	0.00%	0
Agree	15.79%	3
Neither agree nor disagree	21.05%	4
Disagree	36.84%	7
Strongly disagree	26.32%	5
TOTAL		19

Answered: 19 Skipped: 0

Powered by SurveyMonkey

Q10: I prefer teaching in person biochemistry laboratories over online only biochemistry laboratories.

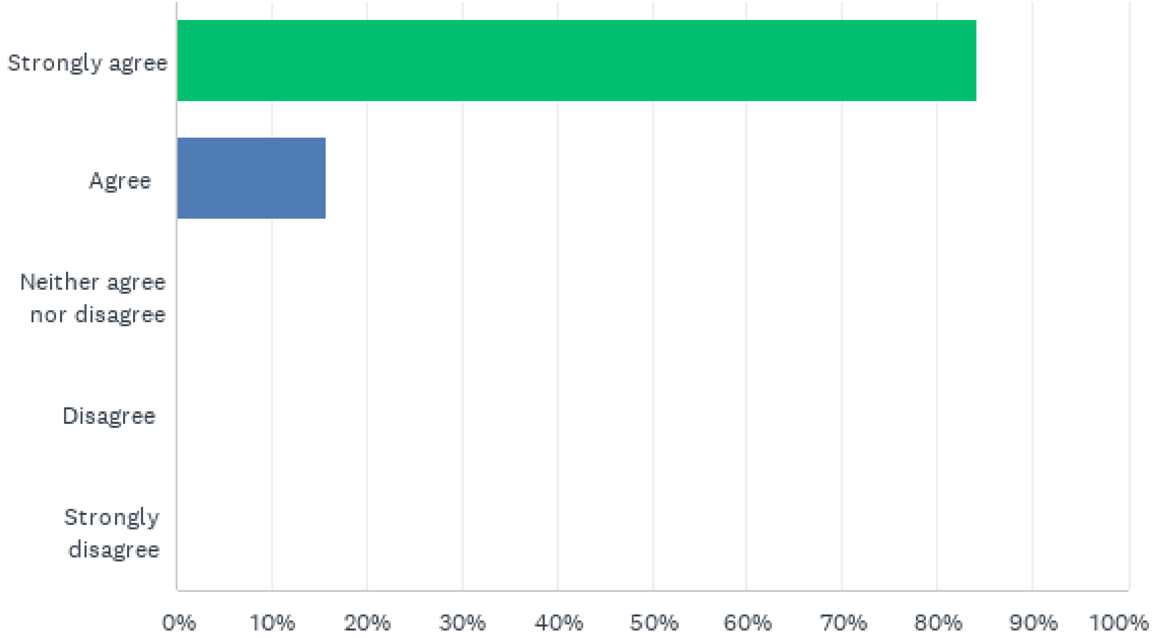

Answered: 19 Skipped: 0

Powered by SurveyMonkey

Q10: I prefer teaching in person biochemistry laboratories over online only biochemistry laboratories.

Answered: 19 Skipped: 0

ANSWER CHOICES	RESPONSES	
Strongly agree	84.21%	16
Agree	15.79%	3
Neither agree nor disagree	0.00%	0
Disagree	0.00%	0
Strongly disagree	0.00%	0
TOTAL		19

Powered by SurveyMonkey

Q11: The pre-made digital biochemistry laboratories that were commercially, or freely, available were sufficient for me to effectively design an online biochemistry laboratory.

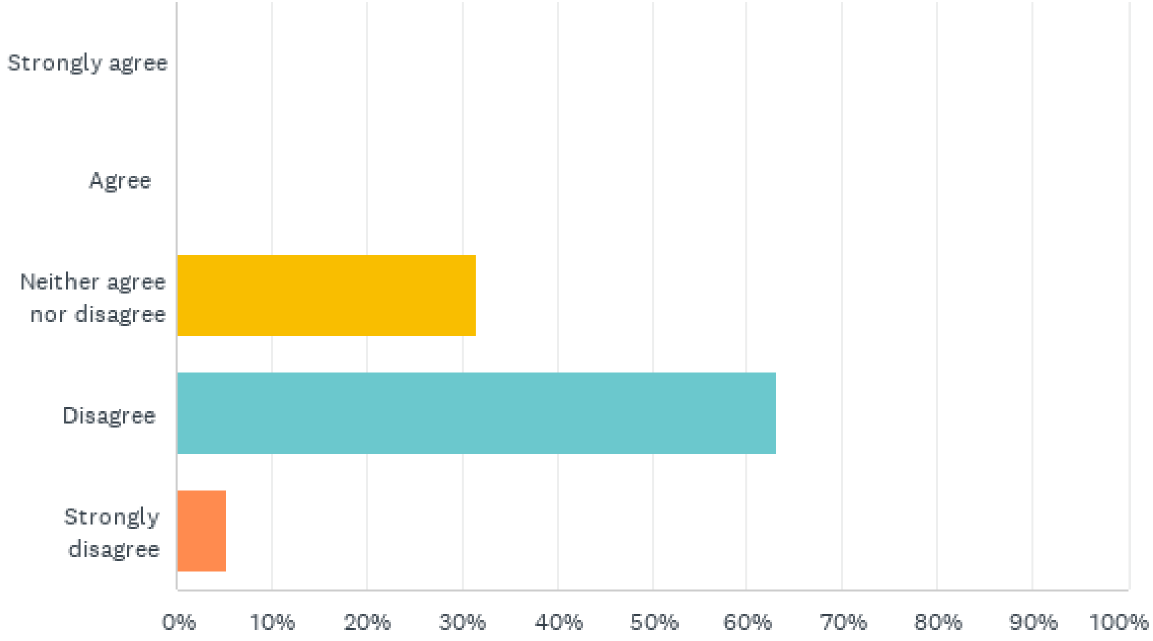

Answered: 19 Skipped: 0

Powered by SurveyMonkey

Q11: The pre-made digital biochemistry laboratories that were commercially, or freely, available were sufficient for me to effectively design an online biochemistry laboratory.

ANSWER CHOICES	RESPONSES	
Strongly agree	0.00%	0
Agree	0.00%	0
Neither agree nor disagree	31.58%	6
Disagree	63.16%	12
Strongly disagree	5.26%	1
TOTAL		19

Answered: 19 Skipped: 0

Powered by SurveyMonkey

Q12: The online only biochemistry laboratory was NOT as effective as in in-person biochemistry laboratory in terms of student learning

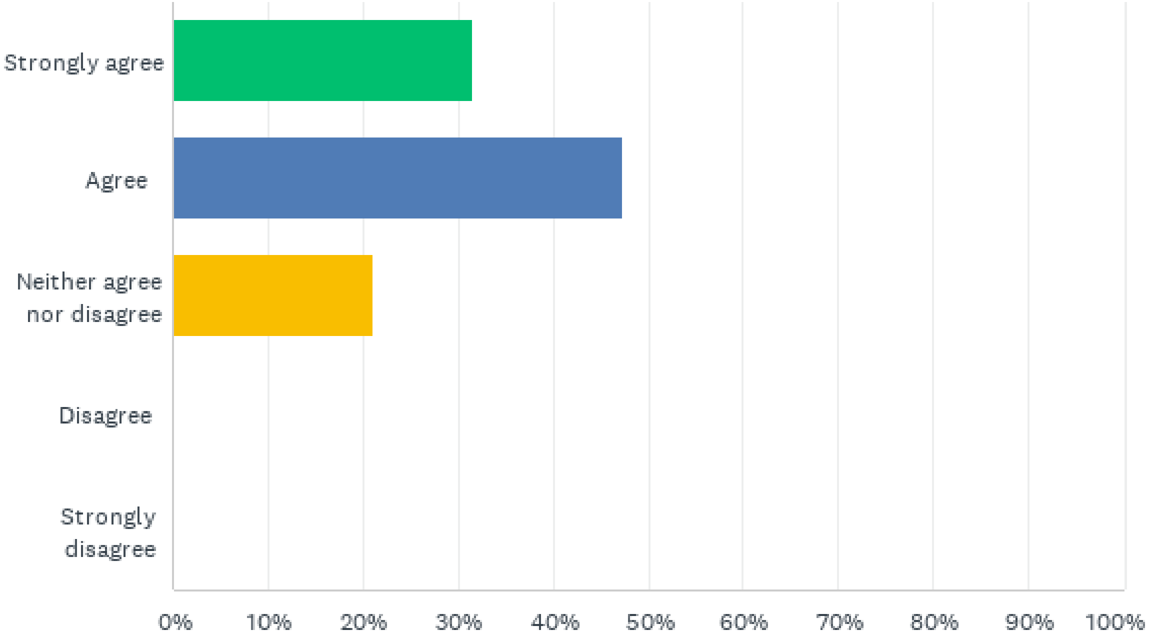

Answered: 19   Skipped: 0

Powered by SurveyMonkey

Q12: The online only biochemistry laboratory was NOT as effective as in in-person biochemistry laboratory in terms of student learning

ANSWER CHOICES	RESPONSES	
Strongly agree	31.58%	6
Agree	47.37%	9
Neither agree nor disagree	21.05%	4
Disagree	0.00%	0
Strongly disagree	0.00%	0
TOTAL		19

Answered: 19 Skipped: 0

Q13: The online only biochemistry laboratory required MORE time and effort from me, the instructor, than an in person biochemistry laboratory.

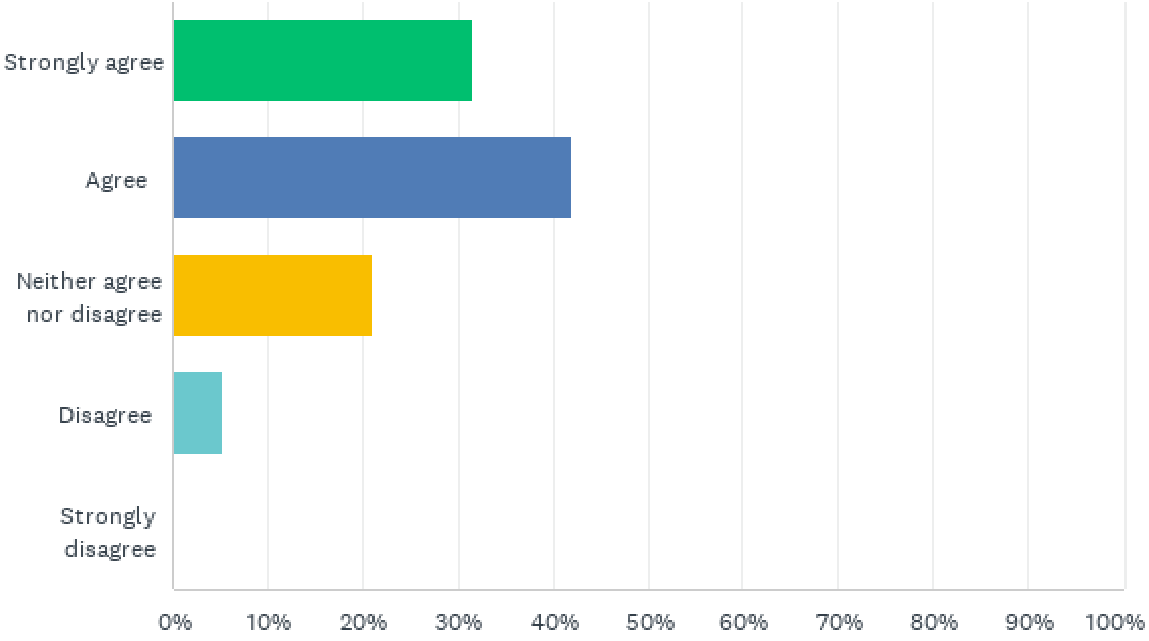

Answered: 19   Skipped: 0

Powered by SurveyMonkey

Q13: The online only biochemistry laboratory required MORE time and effort from me, the instructor, than an in person biochemistry laboratory.

ANSWER CHOICES	RESPONSES	
Strongly agree	31.58%	6
Agree	42.11%	8
Neither agree nor disagree	21.05%	4
Disagree	5.26%	1
Strongly disagree	0.00%	0
TOTAL		19

Answered: 19   Skipped: 0

Powered by SurveyMonkey

Q14: The online only biochemistry laboratory was NOT as effective as in in-person biochemistry laboratory in terms of student engagement and interest

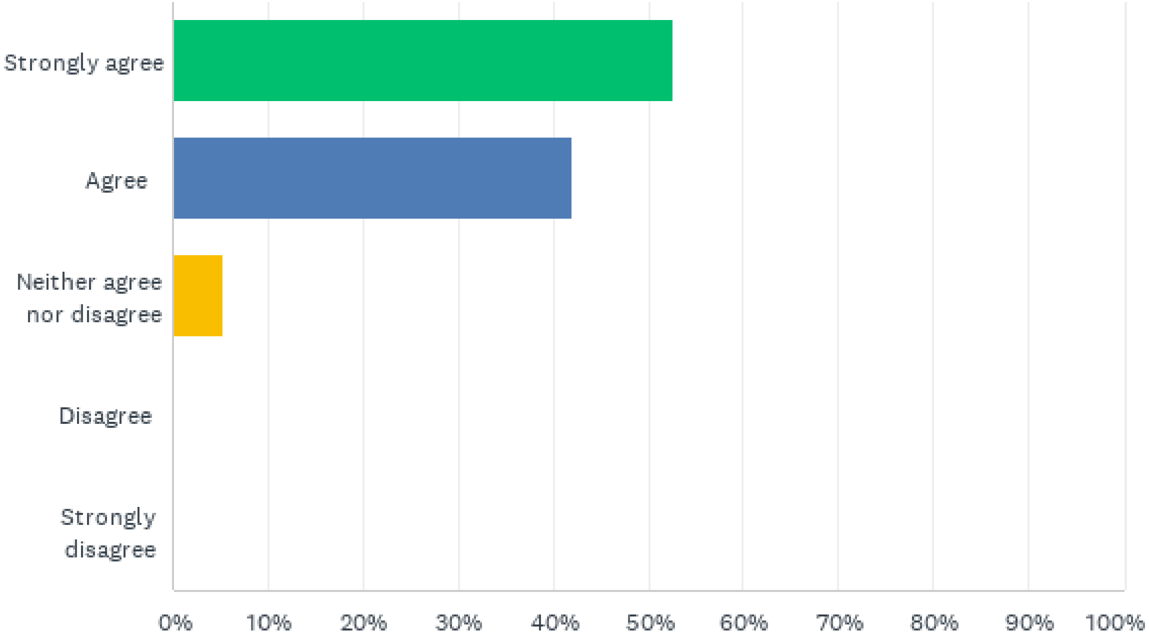

Answered: 19 Skipped: 0

Powered by SurveyMonkey

Q14: The online only biochemistry laboratory was NOT as effective as in in-person biochemistry laboratory in terms of student engagement and interest

ANSWER CHOICES	RESPONSES	
Strongly agree	52.63%	10
Agree	42.11%	8
Neither agree nor disagree	5.26%	1
Disagree	0.00%	0
Strongly disagree	0.00%	0
TOTAL		19

Answered: 19 Skipped: 0

Powered by SurveyMonkey

Q15: My institution provided enough support for the change to the online only biochemistry laboratory.

Answered: 19 Skipped: 0

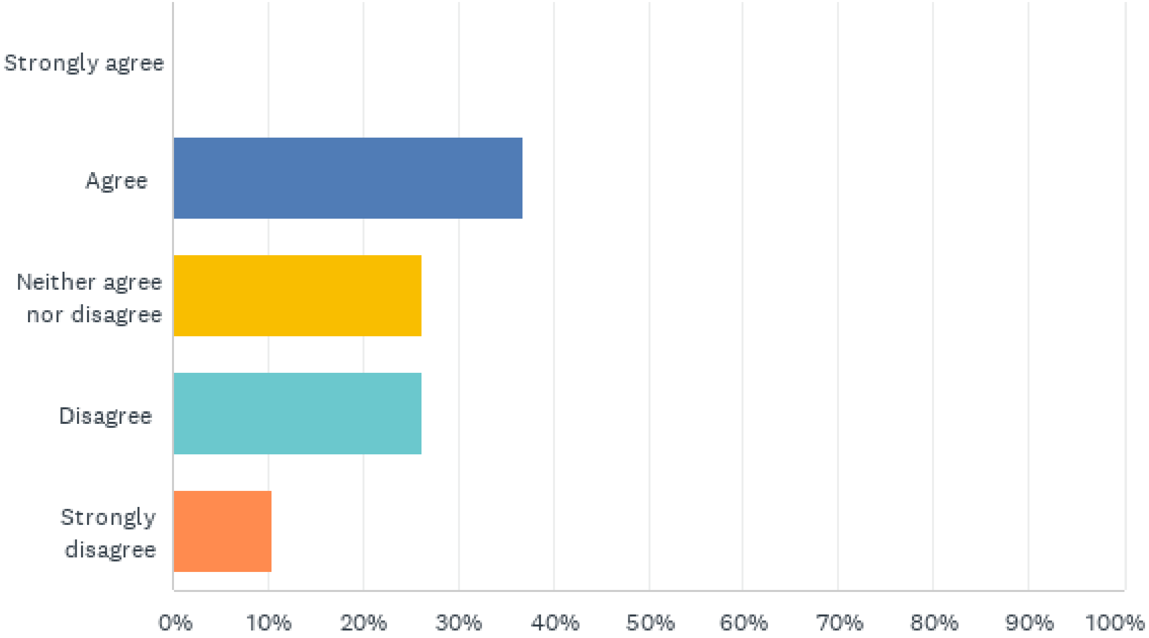

Powered by SurveyMonkey

Q15: My institution provided enough support for the change to the online only biochemistry laboratory.

Answered: 19 Skipped: 0

ANSWER CHOICES	RESPONSES	
Strongly agree	0.00%	0
Agree	36.84%	7
Neither agree nor disagree	26.32%	5
Disagree	26.32%	5
Strongly disagree	10.53%	2
TOTAL		19

Q16: The online only biochemistry laboratory was equivalent or as good as an in-person biochemistry laboratory in terms of student engagement and interest

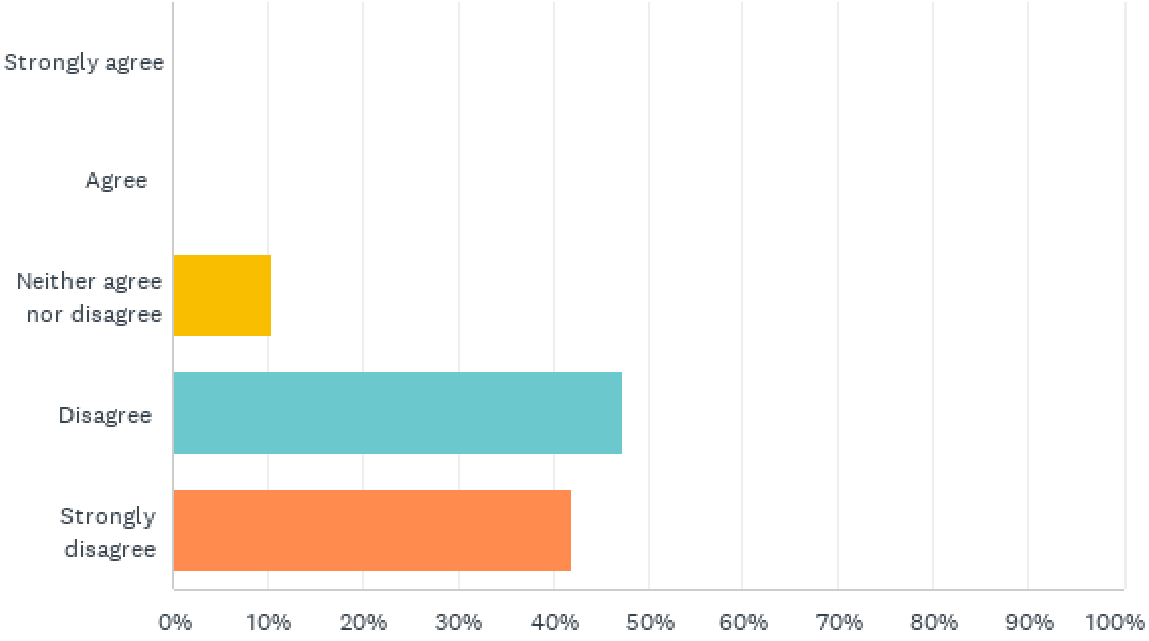

Answered: 19 Skipped: 0

Powered by SurveyMonkey

Q16: The online only biochemistry laboratory was equivalent or as good as an in-person biochemistry laboratory in terms of student engagement and interest

ANSWER CHOICES	RESPONSES	
Strongly agree	0.00%	0
Agree	0.00%	0
Neither agree nor disagree	10.53%	2
Disagree	47.37%	9
Strongly disagree	42.11%	8
TOTAL		19

Answered: 19 Skipped: 0

Powered by SurveyMonkey

Q17: I enjoyed teaching an online only biochemistry laboratory as much as an in person biochemistry laboratory.

Answered: 19   Skipped: 0

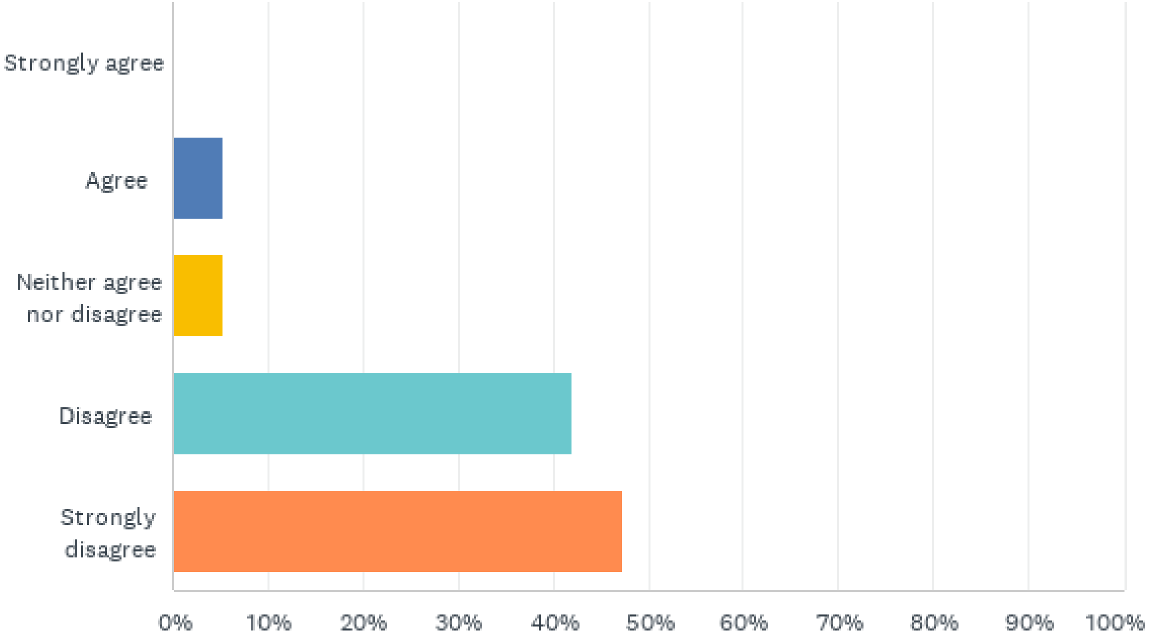

Powered by SurveyMonkey

Q17: I enjoyed teaching an online only biochemistry laboratory as much as an in person biochemistry laboratory.

Answered: 19   Skipped: 0

ANSWER CHOICES	RESPONSES	
Strongly agree	0.00%	0
Agree	5.26%	1
Neither agree nor disagree	5.26%	1
Disagree	42.11%	8
Strongly disagree	47.37%	9
TOTAL		19

Powered by SurveyMonkey

Q18: I will use some online laboratory modules in in-person biochemistry laboratories in the future.

Answered: 19   Skipped: 0

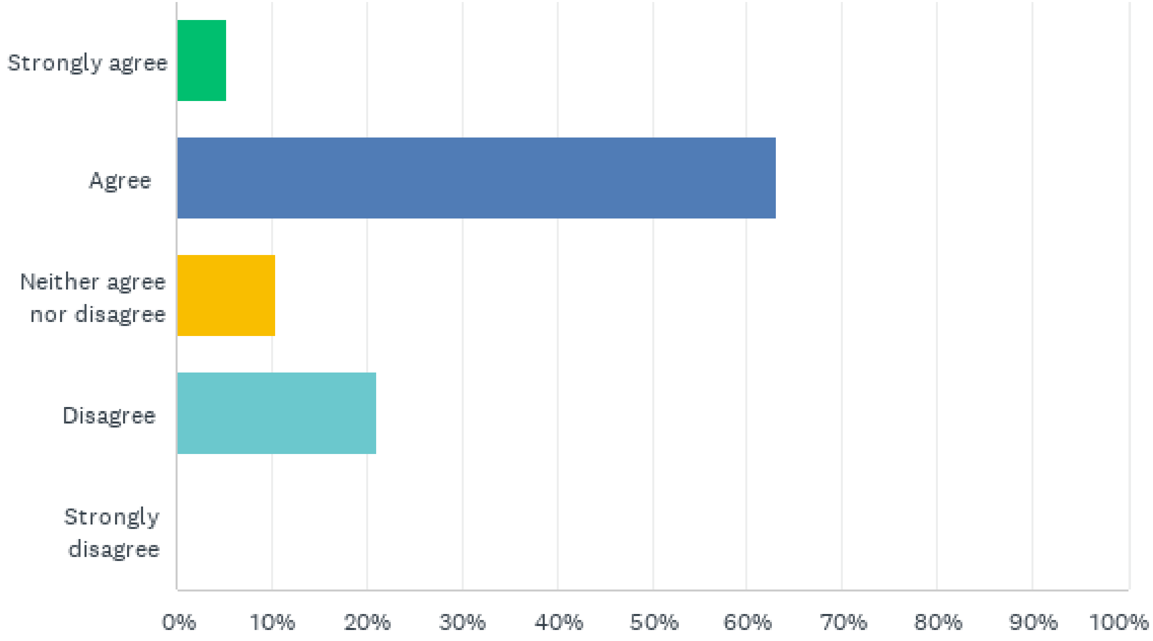

Q18: I will use some online laboratory modules in in-person biochemistry laboratories in the future.

Answered: 19   Skipped: 0

ANSWER CHOICES	RESPONSES	
Strongly agree	5.26%	1
Agree	63.16%	12
Neither agree nor disagree	10.53%	2
Disagree	21.05%	4
Strongly disagree	0.00%	0
TOTAL		19

Q19: I would choose to teach an online biochemistry laboratory course in the future, even if I was not required to do so/it was not needed due to COVID-19 issues.

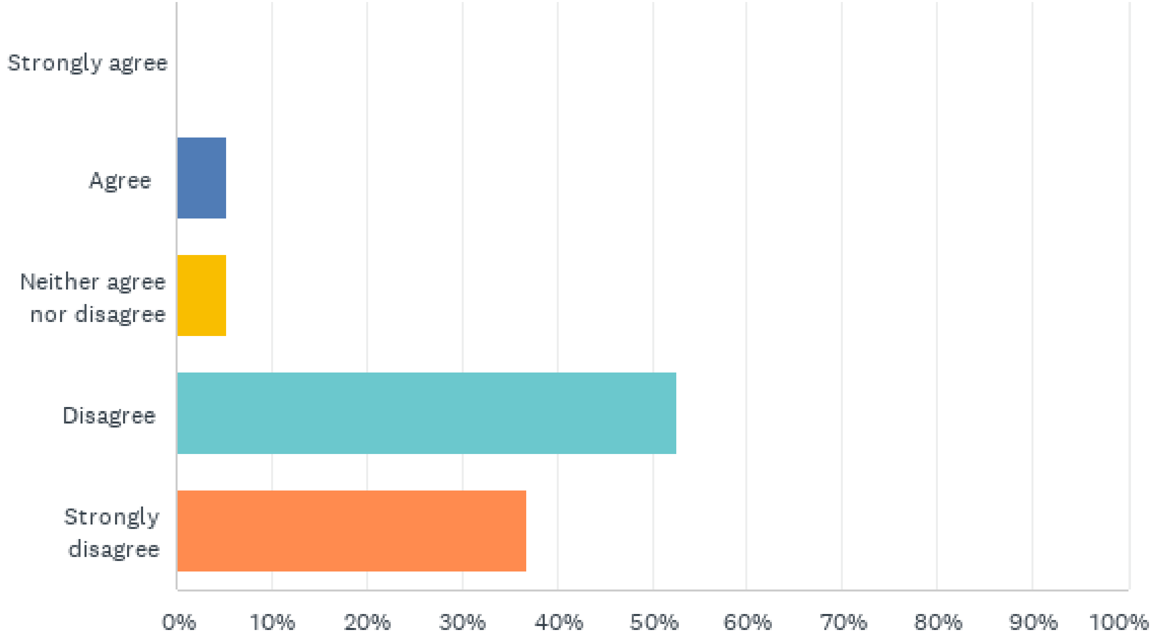

Answered: 19 Skipped: 0

Powered by SurveyMonkey

Q19: I would choose to teach an online biochemistry laboratory course in the future, even if I was not required to do so/it was not needed due to COVID-19 issues.

ANSWER CHOICES	RESPONSES	
Strongly agree	0.00%	0
Agree	5.26%	1
Neither agree nor disagree	5.26%	1
Disagree	52.63%	10
Strongly disagree	36.84%	7
TOTAL		19

Answered: 19   Skipped: 0

Powered by SurveyMonkey

Q20: I had to pre-record or livestream laboratory material for my online only biochemistry laboratory because the commercially, or freely available mate [illegible].

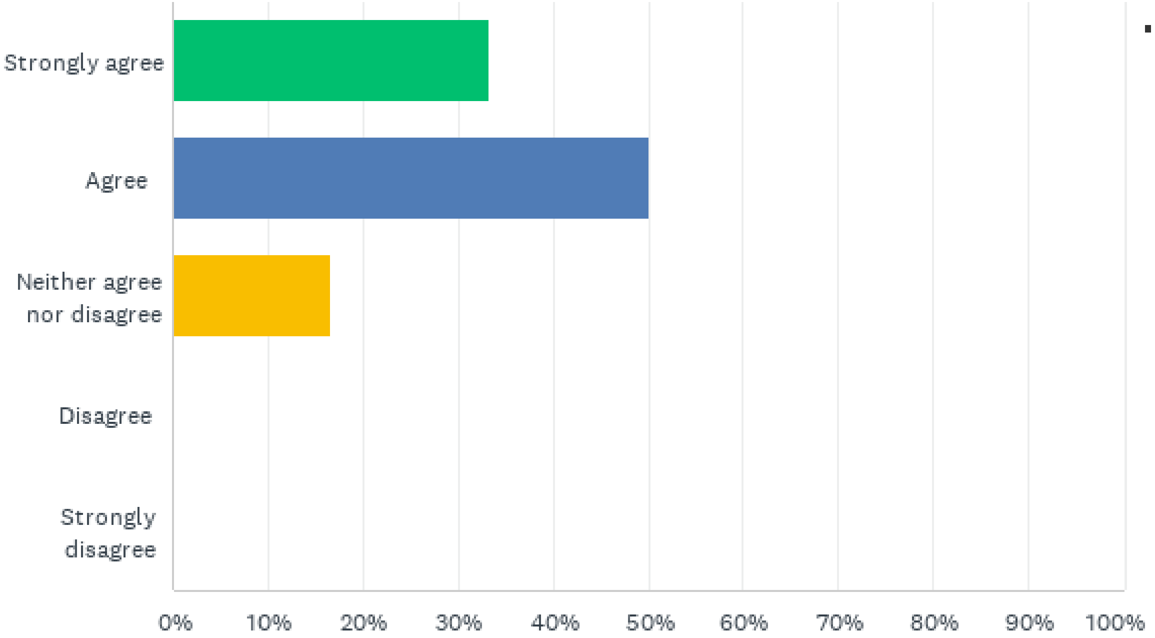

Answered: 18 Skipped: 1

Powered by SurveyMonkey

Q20: I had to pre-record or livestream laboratory material for my online only biochemistry laboratory because the commercially, or freely available material I found was not sufficient in scope and/or quality.

ANSWER CHOICES	RESPONSES	
Strongly agree	33.33%	6
Agree	50.00%	9
Neither agree nor disagree	16.67%	3
Disagree	0.00%	0
Strongly disagree	0.00%	0
TOTAL		18

Answered: 18 Skipped: 1

Powered by SurveyMonkey
